# Dissecting spatial heterogeneity and the immune-evasion mechanism of CTCs by single-cell RNA-seq in hepatocellular carcinoma

**DOI:** 10.1038/s41467-021-24386-0

**Published:** 2021-07-02

**Authors:** Yun-Fan Sun, Liang Wu, Shi-Ping Liu, Miao-Miao Jiang, Bo Hu, Kai-Qian Zhou, Wei Guo, Yang Xu, Yu Zhong, Xiao-Rui Zhou, Ze-Fan Zhang, Geng Liu, Sheng Liu, Ying-Hong Shi, Yuan Ji, Min Du, Nan-Nan Li, Gui-Bo Li, Zhi-Kun Zhao, Xiao-Yun Huang, Li-Qin Xu, Qi-Chao Yu, David H. Peng, Shuang-Jian Qiu, Hui-Chuan Sun, Michael Dean, Xiang-Dong Wang, Wen-Yuan Chung, Ashley R. Dennison, Jian Zhou, Yong Hou, Jia Fan, Xin-Rong Yang

**Affiliations:** 1 Department of Liver Surgery & Transplantation, Liver Cancer Institute, Zhongshan Hospital, Fudan University; Key Laboratory of Carcinogenesis and Cancer Invasion, Ministry of Education, Shanghai, People’s Republic of China; 2 BGI-Shenzhen, Shenzhen, Beishan Industrial Zone, Shenzhen, China; 3 BGI Education Center, University of Chinese Academy of Sciences (UCAS), Shenzhen, China; 4 Shenzhen Key Laboratory of Single-cell Omics, BGI-Shenzhen, Shenzhen, China; 5 State Key Laboratory of Bioelectronics, Southeast University, Nanjing, China; 6 School of Biological Science and Medical Engineering, Southeast University, Nanjing, China; 7 Department of Laboratory Medicine, Zhongshan Hospital, Fudan University, Shanghai, People’s Republic of China; 8 Department of Pathology, Zhongshan Hospital, Fudan University, Shanghai, People’s Republic of China; 9 Dunwill Med-Tech, Shanghai, China; 10 Laboratory of Translational Genomics, Division of Cancer Epidemiology & Genetics, National Cancer Institute Gaithersburg, Rockville, MD USA; 11 Zhongshan Hospital Institute of Clinical Science, Shanghai Institute of Clinical Bioinformatics, Fudan University Center for Clinical Bioinformatics, Shanghai, China; 12 Department of Hepatobiliary and Pancreatic Surgery, University Hospitals of Leicester NHS Trust, Leicester, UK; 13 Zhong-Hua Precision Medical Center, Zhongshan Hospital, Fudan University–BGI, Shanghai, People’s Republic of China

**Keywords:** Liver cancer, Metastasis, Tumour heterogeneity, Tumour immunology

## Abstract

Little is known about the transcriptomic plasticity and adaptive mechanisms of circulating tumor cells (CTCs) during hematogeneous dissemination. Here we interrogate the transcriptome of 113 single CTCs from 4 different vascular sites, including hepatic vein (HV), peripheral artery (PA), peripheral vein (PV) and portal vein (PoV) using single-cell full-length RNA sequencing in hepatocellular carcinoma (HCC) patients. We reveal that the transcriptional dynamics of CTCs were associated with stress response, cell cycle and immune-evasion signaling during hematogeneous transportation. Besides, we identify chemokine CCL5 as an important mediator for CTC immune evasion. Mechanistically, overexpression of CCL5 in CTCs is transcriptionally regulated by p38-MAX signaling, which recruites regulatory T cells (Tregs) to facilitate immune escape and metastatic seeding of CTCs. Collectively, our results reveal a previously unappreciated spatial heterogeneity and an immune-escape mechanism of CTC, which may aid in designing new anti-metastasis therapeutic strategies in HCC.

## Abstract (web summary)

Circulating tumour cells can be useful for monitoring disease progression but how they survive in the circulatory system is unclear. Here, the authors use single-cell sequencing of circulating tumour cells from multiple vascular sites in liver cancer patients and identify genes that may help the cells survive.

## Introduction

Liver cancer is the second leading cause of cancer-related mortality worldwide, and hepatocellular carcinoma (HCC) accounts for 90% of cases^1^. Despite improved surveillance and treatment strategies, the clinical outcome of HCC remains dismal due to the high incidence rates of relapse and metastasis. Hematogenous dissemination is the major route of HCC metastasis^2^, and a thorough investigation into the underlying mechanisms is urgently needed to improve the clinical outcome of HCC patients. Multiple studies have concluded that circulating tumor cells (CTCs) act as the “seeds” for intrahepatic and extrahepatic metastasis (EHM) in HCC^3^. During dissemination, CTCs are exposed to many types of stress exerted by blood microenvironments, including anoikis, shear forces, oxygen/nutrient deprivation, and immune surveillance, all of which must overcome before successful colonization^4^. Thus, targeting CTCs and investigating the changes during their hematogeneous spreading might reveal novel mechanisms for tumor metastasis. Although there has been considerable progress in single-cell RNA sequencing (scRNA-seq) of CTCs^5^, there is a paucity of data regarding CTC plasticity and adaptive mechanisms during the dissemination process.

We and others have previously demonstrated that CTCs dynamically activate the epithelial–mesenchymal transition (EMT) program during hematogeneous transportation^6,7^. Therefore, we hypothesize that CTCs might spatially and temporally modulate their phenotypic characteristics and molecular signaling, during dissemination to survive the inhospitable circulatory microenvironment and colonize distant sites. To explore this question, we establish scRNA-seq profiles of individual CTCs isolated from four key vascular sites along the HCC hematogenous metastatic pathway. Our scRNA-seq data reveal remarkable intravascular and intervascular site heterogeneity among CTCs. By comparing CTCs from neighboring vascular sites, the spatiotemporal transcriptional dynamics associated with cell cycle and immune-evasion signaling are identified along CTCs hematogeneous transportation route. Furthermore, we find CCL5 as an important mediator for CTC immune evasion by the recruitment of regulatory T cells (Tregs) via TGF-β1-p38-MAX signaling.

## Results

### Deciphering distinct expression profiles of CTCs with scRNA-seq

**Fig. 1:**
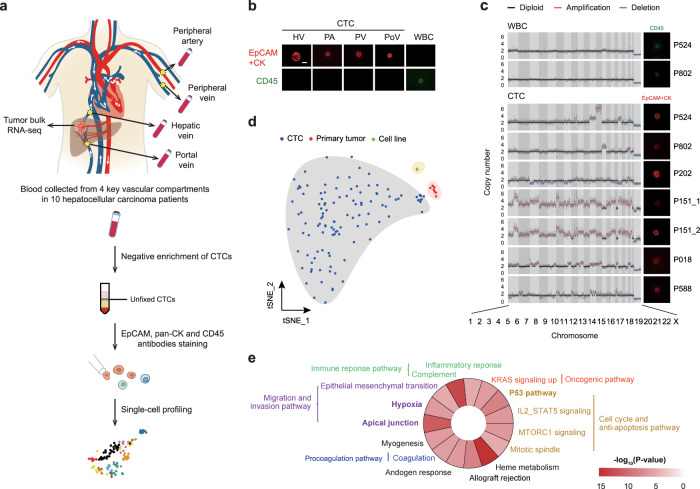
Characterizing differential gene expression among CTCs, primary tumor, and HCC cell lines based on RNA-seq. **a** Overview of the workflow for CTC isolation and single-cell RNA preparation. **b** Representative fluorescence images of CTCs and WBCs labeled with CD45, EpCAM, and CK antibodies; the scale bar is 10 μm. **c** CNV profiling of seven EpCAM^+^ and/or pan-CK^+^ and CD45^−^ CTCs and two CD45^+^ WBC from six patients. The fluorescence images of sequenced CTCs and WBCs are displayed. **d** *t*-SNE plot illustrates the similarity of the expression profiles between CTCs, primary tumors, and HCC cell lines. **e** Pathway enrichment in the Hallmark dataset for upregulated genes in CTCs compared with primary tumors. Hypergeometric test was used to test whether DEGs were overlapped with the gene sets. The Benjamini–Hochberg FDR-controlling method for multiple hypothesis testing were performed.

We used CTC-negative enrichment assay to deplete normal hematopoietic cells from whole-blood samples^8^. Cells positive for EpCAM or pan-CK and negative for CD45 were identified as CTCs^9^. Untagged and unfixed single CTCs were isolated, characterized by immunofluorescence (IF) staining, and micromanipulated for further scRNA-seq (Fig. 1a, b) or single-cell low-pass whole-genome sequencing (LP-WGS; “Methods”). We first characterized EpCAM^+^ and/or pan-CK^+^ and CD45^−^ CTCs isolated by our workflow, using single-cell LP-WGS. All sequenced seven cells from six HCC patients isolated by our workflow that fulfilled with EpCAM^+^ and/or pan-CK^+^ and CD45^−^ criteria showed copy number variation (CNV), while two CD45^+^ white blood cells (WBC) did not (Fig. 1c). These data highlighted that cells separated by our method are malignant cells, which can be considered as CTCs.

A total of 295 CTCs were isolated from four vascular sites (hepatic vein (HV), peripheral artery (PA), peripheral vein (PV), and portal vein (PoV)) in 10 HCC patients before curative resection (Supplementary Data 1), and 119 (40.3%) qualified and were selected for further sequencing and analysis, then 113 (38%) CTC remained after filtering by the amount of sequencing data and detected gene number (“Methods”). The transcriptomes of bulk parental HCCs and paired peritumoral tissues from seven of ten patients, four single cells derived from two HCC cell lines, Hep3B and Huh7, and single leukocytes derived from one patient as control samples were also investigated (Supplementary Data 2). ScRNA-seq inferred CNVs results supported that selected CTCs were tumor cells (Supplementary Fig. 1b and “Methods”). We first visualized the data on a two-dimensional map generated with *T*-distributed stochastic neighbor embedding (*t*-SNE), which revealed that CTCs, primary tumors, and cell lines were separated into three distinct transcriptional patterns (Fig. 1d, and Supplementary Fig. 1c, and “Methods”). Differential expression analysis identified 929 significantly upregulated genes in CTCs compared with primary tumors (Supplementary Data 3 and “Methods”). The most enriched were (i) *NFE2*, members of Cap“n”Collar basic leucine zipper transcription factor (TF) family, which activates the expression of genes contributing to suppress oxidative stress^10^, (ii) *PPBP* has been implicated in guiding the assembly of granulocytes at the early metastatic niche^11^, (iii) the proto-oncogene *GFI1B*^12^, and (iv) the platelet biomarker *ITGA2B*, which indicates the shielding of CTCs with platelets (Supplementary Fig. 1d). Gene set enrichment analysis (GSEA) identified the molecular pathways upregulated in CTCs, including cellular migration and invasion, cell cycle, anti-apoptosis, immune response, and pro-coagulation signaling pathways (Fig. 1e). Genes associated with invasiveness and metastasis, including *ACTB, AMIGO2*, and *S100A4* were upregulated in 67% of CTCs (Supplementary Fig. 1e). Genes encoding proteins involved in energy metabolism reprogramming, including *SLC2A3* and *PDK1*, were highly enriched in most CTCs (Supplementary Fig. 1e). CTCs overexpressed *RAP2B* and *RCHY1* potentiating their DNA damage repair response. Chemokine signaling pathways were also upregulated in CTCs, including *CCL5, CXCL5*, and *CXCL3* (Supplementary Fig. 1e).

### scRNA-seq reveals the spatial transcriptional heterogeneity in CTCs

**Fig. 2:**
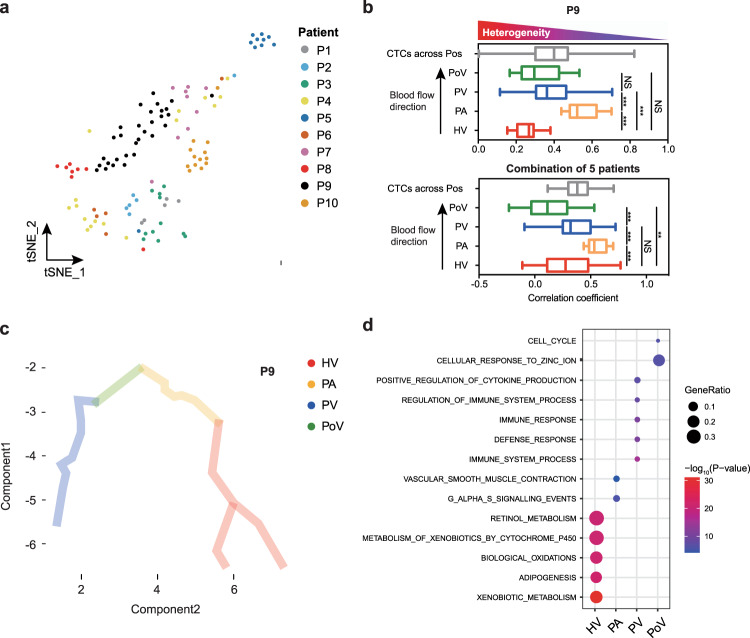
Spatial transcriptional heterogeneity in CTCs. **a** *t*-SNE plot of CTCs from ten patients reveals their interpatient heterogeneity. **b** Pearson correlation analysis showing cell-to-cell variability for CTCs drawn from four vascular sites in patient P9 (upper panel) and five patients combined (lower panel). The two-sided Wilcox test was used. Data are presented using box and whisker plot (median, lower quartile, upper quartile, minimum, and maximum values). *n* = 45 cells in HV; *n* = 12 cells in PA; *n* = 40 cells in PV; *n* = 16 cells in PoV. **P < 0.01, ***P < 0.001. Exact *P* values in upper panel: HV vs PA, *P* = 1.18 × 10^−9^; PA vs PV, *P* = 2.79 × 10^−4^; HV vs PV, *P* = 2.36 × 10^−4^. Exact *P* values in lower panel: HV vs PA, *P* = 2.32 × 10^−3^; PA vs PV, *P* = 7.00 × 10^−4^; PV vs PoV, *P* = 1.50 × 10^−5^; HV vs PoV, *P* = 6.13 × 10^−3^. Bar = median, box plot = quartiles. **c** Pseudotemporal kinetics of CTCs in patients P9. **d** Bubble chart presenting the molecular pathways enriched in CTCs from each vascular sites. Hypergeometric test was used to test whether DEGs were overlapped with the gene sets. The Benjamini–Hochberg FDR-controlling method for multiple hypothesis testing were performed.

The spatial transcriptional heterogeneity in CTC populations during their transportation was then explored. Initially, we found CTCs that isolated from different vascular compartments strongly clustered by the origin of patients, indicating that interpatient heterogeneity is higher than intervascular compartment heterogeneity (Fig. 2a and Supplementary Fig. 2a). Next, we analyzed the intercellular transcriptional diversity of CTCs from spatially four different vascular sites in an individual patient P9. A remarkably heterogeneous CTC population was identified in the liver-efferent HV, while CTCs from post-pulmonary PA exhibited significantly decreased heterogeneity (Fig. 2b, *P* < 0.001). Surprisingly, intercellular transcriptional heterogeneity significantly increased again in the PV and PoV (Fig. 2b, *P* < 0.001). The pattern of spatial heterogeneity of CTCs observed in P9 was further confirmed in other five patients with at least three CTCs detected in two or more vascular sites (Fig. 2b). Analysis using the program Monocle supported the relative higher heterogeneity among HV and PV CTCs, in comparison to PA CTCs. Moreover, pseudotemporal kinetics of CTCs from the HV to the PV through the PA also depicted the anatomical blood flow pathway of disseminating HCC cells (Fig. 2c, Supplementary Fig. 2b–d, and “Methods”).

We next examined the gene profiles of CTCs that specifically expressed in each vascular site. The four vascular site-specific expression profiles of CTCs were enriched for genes related to oxidative phosphorylation/xenobiotic metabolism (HV), GPCR signaling (PA), immune response/cytokine production (PV), and cell cycle (PoV), respectively (Fig. 2d). These results demonstrated that single CTCs from various vascular compartments showed remarkable intravascular and intervascular heterogeneity in their transcriptional profiles, which implied that biophysical cues during their transportation in the bloodstream, including hemodynamic stress, cytokine and immune cell interactions, and oxygen/nutrient deprivation continuously modulated CTC phenotypic diversity^13^.

### Transcriptomic dynamics of CTCs during hematogeneous transportation

**Fig. 3:**
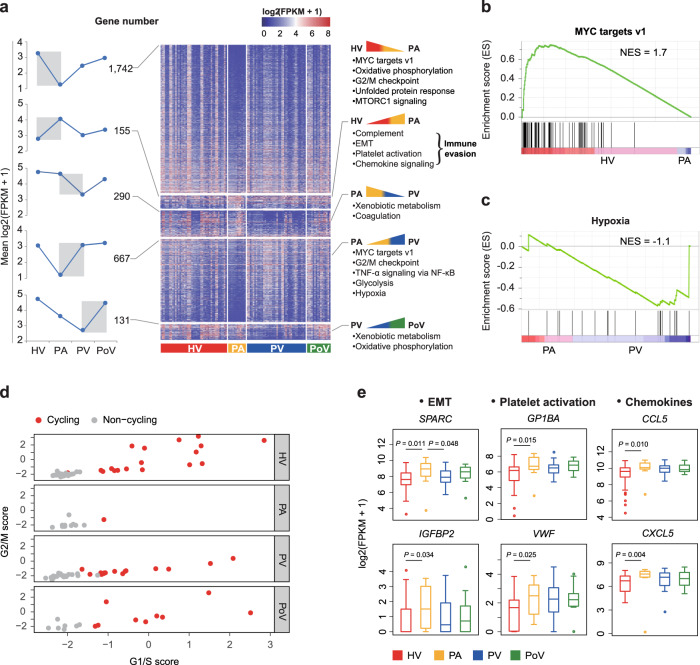
Dynamics of single-CTC transcriptome highlights pathway alterations for stress response and heterogeneity in cell cycle during circulation. **a** Dynamic changes and heat map of differentially expressed genes between neighbor vascular sites (HV vs PA, PA vs PV, and PV vs PoV), with the number of differentially expressed genes indicated at left panel and major signaling pathways enriched (right panel). **b** GSEA of CTCs from HV vs PA for MYC targets v1 pathway. **c** GSEA of CTCs from PA vs PV for hypoxia pathway. **d** Scatterplot indicating cell cycle state of individual CTCs on the basis of G1/S (*x*-axis) and G2/M (*y*-axis) gene sets in different vascular compartments. **e** Box and whisker plots showing expression variance of EMT-related, platelet activation, and chemokine genes in CTCs across four vascular sites. Data are presented as median, lower quartile, upper quartile, minimum, and maximum values. *n* = 45 cells in HV; *n* = 12 cells in PA; *n* = 40 cells in PV; *n* = 16 cells in PoV. The two-sided Wilcox test was used.

The remarkable intercellular heterogeneity observed across four vascular compartments prompted us to investigate the molecular mechanisms underlying CTC plasticity, and the ability to adapt to adverse environmental conditions encountered during dissemination. Indeed, we found 2428 genes with significant changes in expression level between neighboring vascular compartments in differential expression analysis (Fig. 3a, Supplementary Data 4, and “Methods”). Overall, the transcriptional activity in CTCs was reduced in PA, but increased in PV and PoV (Fig. 3a). The most dramatic changes in expression occurred between the HV and PA regions. The downregulated genes were clearly enriched for pathways important for cell growth and proliferation. Genes involved in MYC targets and G2/M checkpoint pathways were upregulated again in some CTCs from venous vascular compartments (Fig. 3b and Supplementary 3a, b). In response to a shortage of oxygen, CTCs in venous vasculatures overexpressed hypoxia-related genes and reprogrammed toward a hypoxic phenotype (Fig. 3c). The considerable gene expression dynamics of cell proliferation pathways between neighboring vascular sites might reflect a spatial heterogeneity of CTC cycling states. To characterize cycling cells more precisely, we used gene signatures that have previously been shown to denote G1/S or G2/M phases in both synchronization and single-cell experiments in cell lines^14^. Cell cycle phase-specific signatures were highly expressed in a subpopulaion of CTCs, distinguishing cycling cells from noncycling cells (Fig. 3d). The proportions of cycling CTCs in HV, PA, PV, and PoV were 40%, 8.3%, 35%, and 56.3%, respectively. We confirmed the existence of cycling and noncycling CTCs by Ki67 staining (Supplementary Fig. 3c).

The upregulated genes from HV to PA were strongly enriched for complement, EMT, platelet activation, and chemokine signaling. Notably, we found that genes implicated in EMT (e.g., *SPARC* and *IGFBP2*), formation of tumor cell-platelet microaggregates (e.g., *GP1BA* and *VWF*) and immunosuppressive chemokines (e.g., *CCL5* and *CXCL5*) showed increased expression from HV to PA, and retained their elevated expression level in PV and PoV (Fig. 3e). CTC subsets expressing platelet biomarkers or showing EMT phenotypes were also reported in previous study of pancreatic cancer^15^. All these biological processes may endow CTCs with immune-evasive advantage to survive encounters with peripheral cytotoxic immune cells, which is of paramount importance for successful CTC-mediated metastasis^13^.

### Immunosuppressive chemokine CCL5 is overexpressed in CTCs

**Fig. 4:**
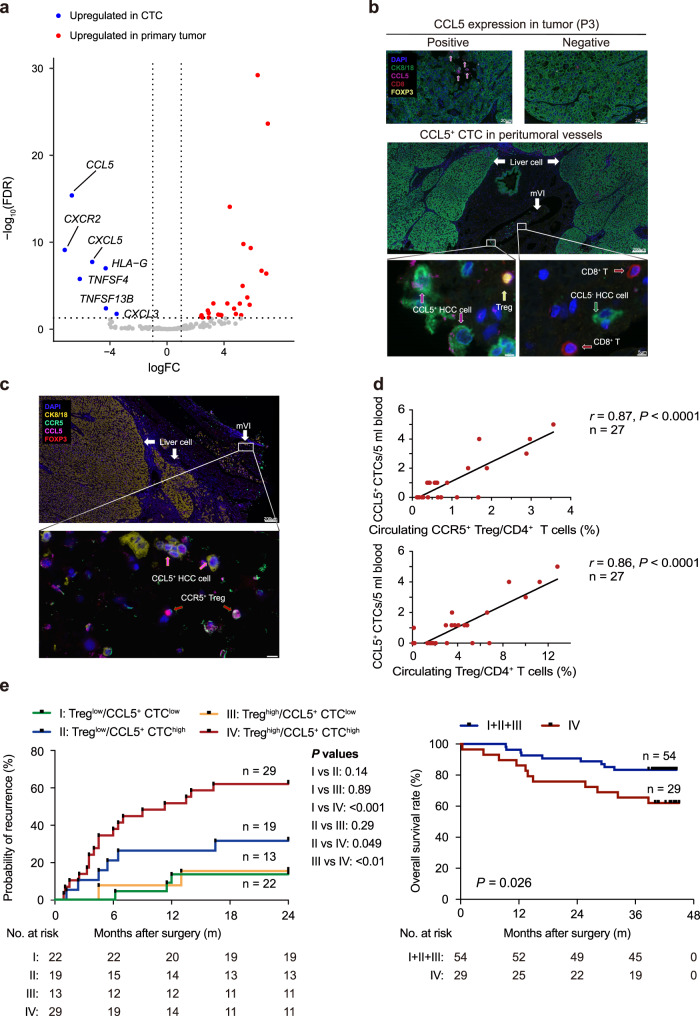
CCL5^+^ CTCs are positively correlated with circulating Tregs and predicted postoperative relapse in HCC patients. **a** Immune-evasion-related genes and cytokines differentially expressed by CTCs and primary tumors. **b** Multiplex immunofluorescence images displaying the expression of CCL5 in primary tumors and CTCs detected in peritumoral microvasculature. mVI, microvascular invasion. The scale bars represent 20 µm, 200 µm, and 5 µm, respectively. **c** Multiplex immunofluorescence images representative of spatial relationship between the CCL5^+^ CTCs and CCR5^+^/FoxP3^+^ Tregs detected in peritumoral microvasculature. The scale bars represent 200 µm and 10 μm, respectively. **d** Scatterplot showing a positive correlation between the number of CCL5^+^ CTCs and the abundance of CCR5^+^ Tregs (CD4^+^, CD25^high^, and CD127^low^) (upper) and total Tregs (lower) in CD4^+^ T cells from HCC peripheral blood (*n* = 27 patients). **e** Kaplan–Meier analysis showing increased probability of early recurrence (left) and decreased overall survival rate (right) in patients with Treg^high^/CTC^high^ in peripheral blood vs the other groups. I: Treg^low^/CCL5^+^ CTC^low^, II: Treg^low^/CCL5^+^ CTC^high^, III: Treg^high^/CCL5^+^ CTC^low^, and IV: Treg^high^/CCL5^+^ CTC^high^. The number of patients at risk for each group is listed below the Kaplan–Meier curve. A two-tailed Student’s *t* test was employed (**d**). Log-rank testing are performed to estimate the prognostic significance (**e**).

To clarify the key gene promoting the immune-evasive ability of CTCs, we next performed differential expression analysis of the most-established signatures linked to cancer immune evasion^16^, and all cytokines between CTCs and primary tumors (Fig. 4a). Among seven highly expressed genes in CTCs, immunosuppressive chemokines^17^ *CCL5* ranks the top one. Then, we performed immunohistochemistry (IHC) to validate the upregulated expression of CCL5 among CTCs in peritumoral microvasculatures from scRNA-seq-matched samples. We found that almost exclusive expression of CCL5 in CTCs compared with primary tumor cells (median, 63% vs 2%, *P* = 0.021, Fig. 4b and Supplementary Fig. 4a). In an independent HCC cohort (validation cohort 1, *n* = 27), CCL5^+^ CTCs were detected in 13 out of 27 patients (48%), and 27 out of 41 CTCs (66%) were CCL5 positive (Supplementary Fig. 4b). Moreover, *CCL5* was significantly upregulated from the HV to PA and remained at a relatively high expression level in the PV and PoV (Fig. 3e), a finding that was also validated by IF assays (Supplementary Fig. 4c).

### CCL5^+^ CTC^high^ and Treg^high^ is associated with poor prognosis

CCL5 has been implicated in recruiting immunosuppressive Tregs to the tumor microenvironment to promote immune escape^17^. We confirmed that CCL5^+^ CTCs were spatially in close proximity to FOXP3^+^ Tregs and were positively correlated with CCR5^+^ Tregs, but not CD3^+^ CD45RO^+/−^ FOXP3^−^ T cells in peritumoral vasculatures, which implied the interaction between the two kinds of cells (Fig. 4c and Supplementary Fig. 4d–f). We next investigated the correlation between CCL5^+^ CTCs and peripheral Tregs in two independent HCC cohorts (Supplementary Fig. 4g). The number of CCL5^+^ CTC was positively correlated with both circulating CCR5^+^ Tregs and total circulating Treg populations in validation cohort 1 (Fig. 4d, *r* = 0.87 and 0.86 respectively; *P* < 0.0001, *n* = 27). Further validation in a cohort of 83 HCC patients (validation cohort 2, Supplementary Data 5) confirmed the positive correlation between CCL5^+^ CTC burden and total Treg populations (Supplementary Fig. 4h, *r* = 0.72, *P* < 0.001). In addition, analysis of two published datasets provided evidence that the expression of *CCL5* was positively correlated with Tregs (*FOXP3*) in HCC tumor tissues and PoV tumor thrombus (Supplementary Fig. 4i, j). These results indicated that Tregs might accompany the dissemination of CCL5^+^ CTCs.

We further investigated the clinical significance of the balance between CCL5^+^ CTC and circulating Tregs using Kaplan–Meier analysis, which revealed a statistically significant shorter time to recurrence (TTR) for patients categorized as Treg^high^/CCL5^+^ CTC^high^ compared to those categorized as Treg^low^/CCL5^+^ CTC^low^, Treg^low^/CCL5^+^ CTC^high^, and Treg^high^/CCL5^+^ CTC^low^ (Fig. 4e). Multivariate analysis indicated that Treg^high^/CCL5^+^ CTC^high^ was the strongest independent prognostic factor for early recurrence (Supplementary Data 6). Patients with Treg^high^/CCL5^+^ CTC^high^ demonstrated a worse overall survival (OS) compared with the rest of patients (Fig. 4e). In addition, TCGA dataset analysis also showed a higher positive correlation coefficient between *CCL5* and *FOXP3* expression in HCC with tumor recurrence compared to cases with good prognosis (Supplementary Fig. 4k, *r* = 0.49 vs 0.13). Taken together, these data provide strong clinical evidence for our hypothesis that CTCs are likely to recruit Tregs via the CCL5 production to facilitate immune escape.

### CCL5 promotes the metastatic potential of CTCs via recruiting Tregs

**Fig. 5:**
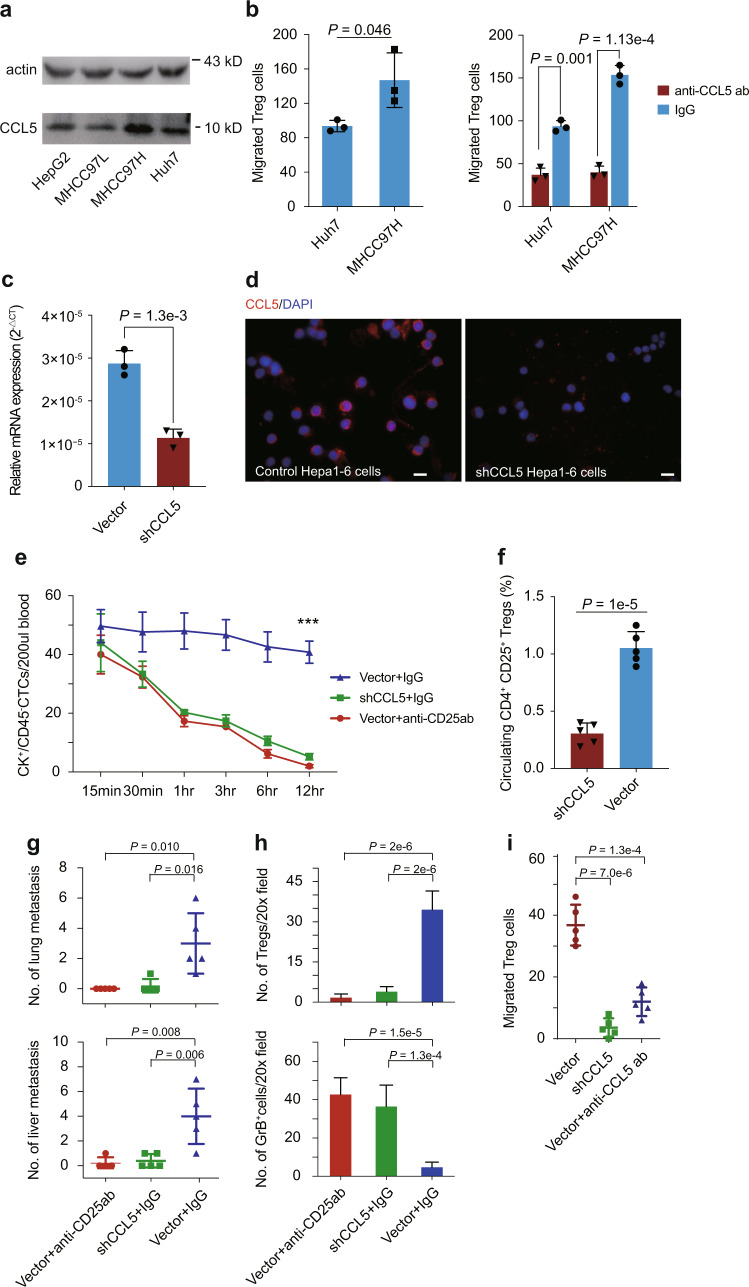
CTCs recruit Tregs via CCL5 expression to generate an immunosuppressive and pro-tumorigenic microenvironment. **a** Expression of CCL5 in HCC cell lines with different metastatic potential. **b** The numbers of migrated Tregs cocultured with supernatant medium of Huh7 or MHCC97H cells without treatment (left panel), and treated with antihuman CCL5-neutralizing antibody or IgG (right panel). **c** Histogram showing relative mRNA expression of *CCL5* in Hepa1–6 cells with CCL5 knockdown or control. **d** Immunofluorescence images of CCL5 expression in Hepa1–6 cells with CCL5 knockdown or control. Scale bar is 10 µm. **e** CTC clearance curves in blood after injection with 5 × 10^6^ Hepa1–6 cells into the tail veins of C57BL/6J mice (*n* = 30 per condition, five mice per time point). **f** Histograms showing the percentage of circulating Tregs in mice from CCL5 knockdown (*n* = 5) and vector control groups (*n* = 5). **g** Scatter plots showing the numbers of lung (upper) and liver metastases (lower) developed under conditions described in **e** (*n* = 5 per group). **h** Histograms showing the numbers of Tregs (upper) and granzyme B^+^ cytotoxic T lymphocytes (lower) in liver metastases under conditions described in **e**. Five independent microscopic field, representing the densest lymphocytic infiltrates, were selected for one liver metastatic tumor each mouse. **i** The comparison of mice Treg migration toward CTCs isolated from vector and shCCL5 Hepa1–6 orthotopic models. CCL5-neutralizing antibody was added to the coculture system to determine the effect on Treg migration (*n* = 5 per group). Comparisons were calculated by two-tailed Student’s *t* test. Data are mean ± SD of three (**b**, **c**) and five (**e**–**g**, **i**) biological replicates, and are representative of two independent experiments. *** represents *P* < 0.001. The exact *P* values at the time point of 12 h in **e**: vector + IgG vs shCCL5 + IgG, *P* = 3.49 × 10^−9^; vector + IgG vs vector + anti-CD25ab, *P* = 1.84 × 10^−9^.

We then examined whether CCL5 could promote the in vitro migration ability of freshly isolated human circulating Tregs. The expression pattern of CCL5 in four HCC cell lines with different metastatic potential was evaluated. A higher protein level of CCL5 was detected in MHCC97H (high metastatic potential) than in HepG2, Huh7, and MHCC97L cells (low metastatic potential; Fig. 5a). We next compared the ability of MHCC97H and Huh7 cells to recruit Tregs in vitro through CCL5. Culture media from MHCC97H demonstrated a significantly improved ability to mobilize Tregs than supernatant from Huh7 cells (Fig. 5b, left panel). Adding antihuman CCL5-neutralizing antibody significantly inhibited the enhanced Treg migratory activity (Fig. 5b, right panel).

To investigate the role of CCL5 in CTC survival and the formation of metastases via Treg recruitment in vivo, we injected 5 × 10^6^ Hepa1–6 murine hepatoma cells with or without knockdown of CCL5 into the tail veins of immune-competent C57BL/6J mice (Fig. 5c, d). Hepa1–6 cells with CCL5 knockdown were cleared more rapidly than empty vector controls within 12 h (Fig. 5e and “Methods”). Empty vector control cells injected into Treg-depleted mice were also cleared significantly more rapidly than the control group (Fig. 5e). We further evaluated the impact of tail-vein injection of CCL5 expressing HCC cells on the number of circulating Tregs. Results indicated that 6 h after tail-vein injection, the CCL5 knockdown group demonstrated a significantly decreased number of circulating Tregs compared with the control group (Fig. 5f and Supplementary Fig. 5a). CCL5-depleted Hepa1–6 cells exhibited a dramatically impaired potential to generate lung and liver metastases (Fig. 5g and Supplementary Fig. 5b). After quantifying the number of Tregs and granzyme B^+^ (GrB^+^) cytotoxic T lymphocytes (CTLs) in metastatic liver tumors, we found that CCL5 downregulation significantly abrogated Treg recruitment and promoted infiltration of activated CTLs (Fig. 5h and Supplementary Fig. 5c). Finally, we established orthotopic HCC mouse models using shCCL5 and vector control Hepa1–6 cells. CCL5 knockdown significantly reduced metastatic growth in the lung (Supplementary Fig. 5d) and CTC numbers in blood (Supplementary Fig. 5e). CTCs were isolated from orthotopic models for in vitro coculture experiment with mouse Tregs. Results indicated that CTCs from shCCL5 group demonstrated a significantly impaired Treg cell recruitment ability compared to the control group (Fig. 5i). Adding anti-CCL5 antibodies could effectively block the ability of CTCs from control group to recruit Tregs in vitro (Fig. 5i). The findings from our in vitro and in vivo assays demonstrate that CTCs exploit a self-defensive mechanism by recruiting Tregs via CCL5 to establish a metastatic-favorable microenvironment during hematogenous transportation.

### *CCL5* induction is mediated through p38-MAX signaling

**Fig. 6:**
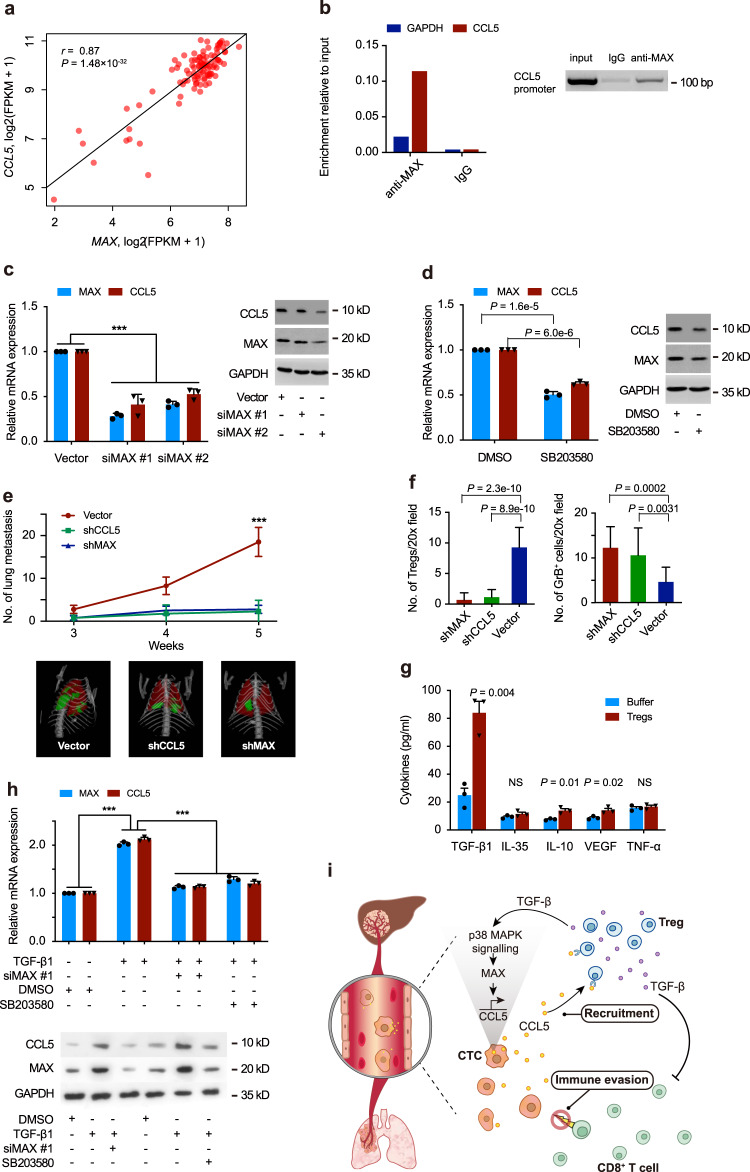
CCL5 induction is mediated through p38-MAX pathway. **a** Scatterplot showing a high correlation between *CCL5* and *MAX* expressed in CTCs based on scRNA-seq. A two-tailed Pearson correlation test was employed. **b** ChIP assays showing direct binding of *MAX* to the *CCL5* gene promoter in MHCC97H cells, using IgG as negative control. **c** Relative expression of *MAX* and *CCL5* at mRNA (left) and protein level (right) in MHCC97H cells transfected, respectively, with MAX siRNAs and vector, and **d** treated with p38 inhibitor SB203580 or DMSO control. **e** The number and representative images (metastatic nodules in green) of lung metastases detected by microCT in different time points after tail-vein injection of 5 × 10^6^ Hepa1–6 cells with three different conditions in C57BL/6J mice (*n* = 5 per group, upper panel). **f** Histograms showing the numbers of Tregs (left) and granzyme B^+^ cytotoxic T lymphocytes (right) in lung metastases from mice treated, as described in **e**. Five independent microscopic field, representing the densest lymphocytic infiltrates, were selected for one lung metastatic tumor each mouse. **g** Comparing levels of Treg-derived cytokines, including TGF-β1, IL-10, IL-35, VEGF, and TNF-α in culture medium from Huh7 cells cocultured with or without Tregs: error bars. **h** The mRNA (upper) and protein (lower) levels of *MAX* and *CCL5* in MHCC97H cells treated with TGF-β1, MAX siRNA, P38 inhibitor, and DMSO, respectively. **i** Schematic illustration of immune-escape mechanism by which CTCs acquire the ability to recruit immunosuppressive Treg cells via a positive feedback loop of TGF-β1-p38-MAX-CCL5 signaling, consequently promoting the formation of a metastatic-favorable microenvironment in the bloodstream and secondary organs. Comparisons were calculated by two-tailed Student’s *t* test (**c**–**h**). Data are mean ± SD of three biological (**c**, **d**, **g**, **h**) and five biological (**e**) replicates, and are representative of two independent experiments. *** represents *P* < 0.001. The exact *P* values for comparison of MAX expression in **c**: vector vs siMAX#1, *P* = 2.00 × 10^−6^; vector vs siMAX#2, *P* = 8.96 × 10^−4^. The exact *P* values for comparison of CCL5 expression in **c**: vector vs siMAX#1, *P* = 8.00 × 10^−6^; vector vs siMAX#2, *P* = 1.51 × 10^−4^. The exact *P* values at the time point of 5 weeks in **e**: vector vs shCCL5, *P* = 2.8 × 10^−4^; vector vs shMAX, *P* = 1.1 × 10^−4^. The exact *P* values for comparison of MAX expression in **h**: DMSO vs TGF-β1, *P* = 2.00 × 10^−6^; DMSO vs TGF-β1 + siMAX#1, *P* = 8.00 × 10^−6^; DMSO vs TGF-β1 + SB203580, *P* = 5.00 × 10^−5^. The exact *P* values for comparison of CCL5 expression in **h**: DMSO vs TGF-β1, *P* = 2.00 × 10^−6^; DMSO vs TGF-β1 + siMAX#1, *P* = 5.00 × 10^−6^; DMSO vs TGF-β1 + SB203580, *P* = 1.8 × 10^−5^.

To identify a candidate regulator of *CCL5* induction, we first screened for TFs whose expression was positively correlated with *CCL5* levels in our single-cell dataset. Among 43 TFs upregulated in CTCs compared with primary tumors, 33 TFs were positively associated with *CCL5* expression. MYC-associated factor X (*MAX*), the top-ranked TF (*r* = 0.86, *P* = 2.04 × 10^−35^, Fig. 6a and Supplementary Data 3), is also one of the TFs that is able to bind to the *CCL5* gene enhancer as annotated by GeneHancer^18^. In patient-derived CTCs, IF analysis demonstrated nuclear co-expression of MAX and CCL5 (Supplementary Fig. 6a). The HCC dataset from TCGA confirmed the positive correlation between *MAX* and *CCL5* (Supplementary Fig. 6b). In addition, KEGG analysis of the differentially upregulated gene set in CTCs showed significant enrichment for p38 MAPK signaling (Supplementary Data 3). Multiple components of p38 MAPK signaling, including *TAOK1, TAOK2, MAP2K3, PLA2G4A*, and *MAX* were overexpressed in CTCs, indicating increased pathway activation in CTCs (Supplementary Fig. 6c). Thus, our single-cell data highlights the role of p38-MAX signaling activation in the control of *CCL5* transcription in CTCs.

To further confirm whether the p38 MAPK pathway and *MAX*-mediated *CCL5* induction, we first performed chromatin immunoprecipitation (ChIP) and luciferase reporter gene assays. ChIP assay demonstrated a direct binding of MAX to the *CCL5* gene promoter in MHCC97H cells (Fig. 6b). Luciferase reporter gene assay also indicated that *CCL5* transcription was dependent on the ability of MAX-specific binding to the *CCL5* promoter and the activation of p38 signaling (Supplementary Fig. 6d). We next treated MHCC97H cells with siRNAs against *MAX* or p38 inhibitor SB203580 and determined the expression level of downstream *CCL5*. Both knockdown of *MAX* with two independent siRNAs and blocking the p38 MAPK pathways by its inhibitor significantly suppressed *CCL5* expression, which was validated by western blot (Fig. 6c, d). Moreover, our in vivo study demonstrated that tumor growth and metastatic potential of Hepa1–6 cells was significantly repressed by MAX or CCL5 depletion in immune-competent mice in comparison to that of the vector control (Fig. 6e and Supplementary Fig. 6e, f). We further used IHC assay to quantify the number of FoxP3^+^ Tregs and GrB^+^ CTLs in lung metastatic nodules. Both MAX and CCL5 knockdown were associated with a substantial reduction in Tregs and an increased infiltration of activated CTLs (Fig. 6f and Supplementary Fig. 6g).

### Treg-derived TGF-β1 induces *CCL5* production via p38-MAX signaling

The finding that the *CCL5* expression in CTCs was dynamically regulated during circulation combined with previous evidence of increased Treg pools in the peripheral blood of HCC patients^19^, led us to consider a possible role for a Treg-rich blood microenvironment in *CCL5* induction of CTCs. To address this hypothesis, we first performed gene expression profiling in Huh7 cells cocultured with or without freshly isolated peripheral Tregs of HCC patients. The upregulated gene set in Treg-cocultured Huh7 cells was associated with several biological processes and pathways, including immune response, transcription regulation, and cytokine–cytokine receptor interaction (Supplementary Fig. 6h, i). We have verified the top three differentially expressed cytokines/chemokines in Huh7 HCC cells cocultured with Tregs, including IL15, CCL5, and LIF (Supplementary Fig. 6j). Blocking IL15 and LIF had no impact on Treg migration, while neutralizing CCL5 showed significant inhibition of Treg migration (Supplementary Fig. 6k). Moreover, TF *MAX* was also identified among upregulated gene sets. To further understand how Tregs contribute to induce *CCL5* expression in HCC cells, the five most-established Treg-derived cytokines, including TGF-β1, IL-10, IL-35, VEGF, and TNF-α were examined in culture medium from Huh7 cells cocultured with or without Tregs, using cytometric bead array (CBA). Results indicated that TGF-β1 was the most abundant cytokine in Treg-cocultured medium (Fig. 6g). We then tested whether TGF-β1 could induce the expression of MAX and CCL5. As shown in Supplementary Fig. 6l, the expression levels of both CCL5 and MAX were increased following TGF-β1 treatment in a time-dependent manner. Luciferase reporter gene assay indicated that *CCL5* promoter showed a transcriptional response to TGF-β1 (Supplementary Fig. 6m). We also observed that the depletion of MAX or inhibiting p38 signaling significantly blocked the ability of TGF-β1-induced CCL5 expression in MHCC97H cells (Fig. 6h). Altogether, these lines of evidence suggested that peripheral Tregs induced CCL5 expression by secretion of TGF-β1 acting through p38-MAX signaling.

## Discussion

CTCs are heterogeneous in nature. Recent multi-marker characterization and downstream molecular analysis revealed tremendous biological heterogeneities of CTCs and their clinical significance. MAGE3/Survivin/CEA expressing CTCs were predictors for response of cryosurgery^20^, while pERK+/pAkt− or PD-L1 + CTCs were respectively correlated with therapeutic response to sorafenib or anti-PD-1 therapy in HCC patients^21,22^. EMT is often seen as a prerequisite to cancer dissemination. Recent studies demonstrated that variable states of epithelial, mesenchymal, or hybrid EMT exist among CTCs, suggesting a continuum and heterogeneous EMT spectra in CTCs^7,23^. Moreover, emerging studies have revealed the transcriptional heterogeneity among individual CTCs and their metastatic mechanisms by using single-cell omics technologies^24^. Therefore, dissecting CTC heterogeneity especially at single-cell resolution may provide unique insights into tumor heterogeneity and mechanisms of cancer metastasis.

Like the heterogeneity of the primary tumor, the heterogeneity of CTC can also be defined in both “space and time”^25^. Recent studies have revealed substantial temporal heterogeneity in CTC phenotypes during anticancer treatments^6,26^. However, the spatial heterogeneity of CTCs within anatomically distinct regions of the human circulatory system has been largely ignored. Our data demonstrated that the inter-CTC heterogeneity varied significantly among different vascular compartments. A remarkably heterogeneous CTC population was identified in liver-efferent vessels (HV), supporting the hypothesis that CTCs were shed randomly from several spatially distinct regions of primary tumors. In the PA, the intercellular heterogeneity of CTCs decreased significantly, implicating a selection process where smaller and more deformable cells preferentially pass the pulmonary capillary filter. Interestingly, the intercellular heterogeneity of CTCs increased again in PV and PoV. Given the complexity of the bloodstream microenvironment, CTCs are exposed to various biophysical cues during peripheral circulation, including flow-based shear stresses, loss of anchorage, and interaction of cytokines and immune cells^16,27^. The flow-based shear stresses and loss of anchorage will promote EMT in CTCs^7^. After tumor cells entering the bloodstream, they inevitably collide with platelets. A common way of physical protection of CTCs is the formation of tumor cell-platelet microaggregates, which results in the physical shielding of CTCs from the damage of blood shear force and from attacks of immune cells. These microaggregates are produced rapidly after tumor cells entering the bloodstream as tumor cells efficiently induce activation of platelets and coagulation factors^13^. Moreover, in response to the challenge of immune surveillance, CTCs may elevate the expression of immunosuppressive chemokines, such as CCL5 and CXCL5, which facilitate the recruitment of Tregs and neutrophils, respectively. These immunosuppressive cells will escort the dissemination of CTCs by diminishing antitumor immune response. To restore cellular homeostasis, CTCs might activate adaptive stress response pathways that not only increase stress tolerance, but may also contribute significantly to their phenotypic diversity. Accordingly, our data showed that the biological processes involved in cell cycling, immune response, regulation of cytokine production, and response to stimuli were specifically enriched in PV CTCs. Thus, CTCs in tumor efferent vessels may represent intratumoral heterogeneity, whereas the transcriptional diversity observed in peripheral CTCs informs their adaptation-related evolution.

Once CTCs leave the protective immunosuppressive tumor microenvironment, they are outnumbered by peripheral immune effector cells. The successful evasion of immune-mediated killing is critical for CTC survival and dissemination. The present work sheds light on how CTCs create an immunosuppressive milieu by hyperactivation of the p38-MAX-CCL5 axis for the recruitment of Treg cells (Fig. 6i). Consistent with recent reports^16^, our scRNA-seq data showed that CTCs exploit a variety of immune-evasion strategies, including EMT, platelet-CTC aggregates, and the production of immunosuppressive chemokines. Notably, the chemokine *CCL5* ranked as the top differentially upregulated transcriptome related to immune evasion, implicating its pivotal role in CTC immune escape. Although evidence indicates that CCL5 promotes tumor growth by increasing Treg infiltration into the tumor microenvironment^17^, our current findings suggested that overexpression of CCL5 in CTCs drives the recruitment of Tregs to support CTC intravascular survival and metastatic colonization. We further dissected the molecular mechanism involved in the transcriptional induction of CCL5. We have shown that activation of p38 signaling triggers CCL5 induction, mediated through the transcriptional regulator MAX. Given that p38 signaling is a well-known stress-activated pathway, we postulate that p38-dependent induction of CCL5 might be partly a cell-intrinsic response to the stresses encountered during CTCs dissemination^28^.

CTCs gradually upregulated the CCL5 expression during their transit from the tumor efferent vessel to peripheral vessels, which led us to consider whether the peripheral Tregs could be the extrinsic factor inducing CCL5 expression in CTCs. When cocultured with HCC cells, circulating Tregs secreted TGF-β1 and effectively boosted CCL5 expression in HCC cells. Using a pharmacological inhibitor of p38, we have identified p38 signaling as the pathway downstream of TGF-β1 stimulation. Furthermore, we found that CCL5 induction by TGF-β1 was a MAX-dependent process since knockdown of MAX greatly repressed TGF-β1-induced CCL5 expression. The intrinsic and extrinsic signals are synergistic, and converge to enhance production of immunosuppressive chemokine CCL5 from CTCs and recruitment of Treg cells.

The limitation of this study is relatively low numbers of CTCs sequenced for each vascular site. More than half of the isolated CTCs failed to pass quality control for scRNA-seq. The experiment for CTC scRNA-seq contains complex and multistep procedures. From CTC enrichment, characterization, single-cell manipulation to sequencing preparation, and many factors that may compromise the RNA quality of single CTCs. This is the reason why it is extremely difficult to harvest a large number of single-CTC transcriptomic data qualified for downstream bioinformatics analysis. Therefore, a future workflow optimized for single-CTC RNA-seq is urgently needed.

Our findings raise the possibility of selectively inhibiting Tregs to trigger the immunologic elimination of latent metastatic cells and immunotherapy targeting Tregs is a promising antitumor strategy^29^. However, unselective depletion of Tregs may cause severe autoimmune disorders. As CCL5 has been identified as a key chemokine produced by CTCs to recruit Tregs clinically it would be safer to develop anti-CTC immunotherapy via blocking CCL5 or CCR5/CCR4. Moreover, although the gradually increased expression of CCL5 during dissemination is an important pro-survival mechanism for CTCs in the bloodstream, it is also their achilles heel for designing new anti-metastasis therapeutic strategies of HCC. Targeting CCL5 or CCR5/CCR4 may provide an opportunity to annihilate CTCs within blood vessels, preventing their arrival at distant organs. This may effectively reduce the risk of developing distant metastasis in HCC. Our discovery of the CTC-CCL5-Tregs axis that induces an immunosuppressive microenvironment in the bloodstream, and distant organs holds translational significance for a therapeutic strategy to suppress CTC survival and metastatic seeding.

## Methods

### Patients and specimens

From September 2013 to June 2016, a total of 120 patients newly diagnosed with HCC undergoing curative resection were recruited to this study. An overview of patient groups is shown in Supplementary Fig. 1a. The entry criteria were (1) definitive pathological or radiological diagnosis according to American Association for Study of Liver Disease guidelines, (2) no EHM at the time of diagnosis, and (3) no prior anticancer treatment^30^. Tumor stage was determined according to the Barcelona Clinic Liver Cancer staging system and China staging system for liver cancer (2017 edition)^1^, and tumor differentiation was defined according to the Edmondson grading system. HCC patient demographics are provided in Supplementary Data 1 and 5. Patients with histories of other solid tumors were excluded.

For ten patients recruited for CTCs scRNA-seq experiments (age ranged from 51–66 years, all males), 20 ml blood was drawn from the following locations: PV (the antecubital fossa) and PA (radial artery) immediately prior to preoperative anesthesia, and HV and PoV intraoperatively before the primary HCC tumor was disturbed. HV and PoV blood samples were obtained from a direct venous puncture after limited mobilization of portal triads and coronary ligament to expose the HV and PoV during operation, respectively (Fig. 1a). During these manipulations, we avoided handling the tumor itself. Frozen primary tumor tissues from seven of ten patients were sectioned, macrodissected for >70% tumor content, and subjected to RNA extraction. For each patient enrolled in validation cohort 1 and 2, 5 ml peripheral venous blood was collected and used for CTC characterization and 1 ml blood for Treg detection.

Ethical approval for the use of human subjects was obtained from the Research Ethics Committee of Zhongshan Hospital in compliance with the ethical guidelines of the 1975 Declaration of Helsinki, and informed written consent was obtained from each patient.

### CTCs isolation and characterization

All blood samples were processed by RosetteSep Human CD45 Depletion Cocktail (STEMCELL Technologies) to deplete normal blood cells and enrich CTCs. For scRNA-seq, unfixed CTCs were stained with a PE-labeled EpCAM antibody (130-098-113, diluted 1:20; Miltenyi Biotec, Bergisch Gladbach, Germany), an Alexa-594-labeled pan-CK antibody (628606, diluted 1:20; BioLegend, San Diego, CA, USA), an Alexa-594-labeled CK19 antibody (ab203443, diluted 1:20; Abcam, Cambridge, UK), and a FITC-labeled CD45 antibody (304006, diluted 1:20; BioLegend). EpCAM^+^ and/or CK^+^ and CD45^−^ cells were defined as CTCs (Fig. 1b). Single CTCs from each patient were transferred under direct microscopic visualization to individual PCR tubes with a robotic micromanipulator (Eppendorf, Hamburg, Germany) for subsequent single-cell RNA preparation. The above process was done within 2.5 h (Fig. 1a, b). For CTC multiplex immunofluorescent staining in validation cohorts, CTCs were fixed after enrichment and stained with PE-labeled EpCAM antibody (130-098-113, diluted 1:50; Miltenyi Biotec, Bergisch Gladbach, Germany), an Alexa-594-labeled pan-CK antibody (628606, diluted 1:50; BioLegend, San Diego, CA, USA), an Alexa-594-labeled CK19 antibody (ab203443, diluted 1:50; Abcam, Cambridge, UK), a APC-labeled CD45 antibody (304012, diluted 1:50; BioLegend), or Alexa-488-labeled CCL5 (IC278G-100UG, diluted 1:50; R&D Systems, Minneapolis, MN, USA) to characterize CCL5 expression in CTCs.

### RNA sequencing of single cells and tissues

Total RNAs from tissues were extracted by a RNeasy plus mini kit (Qiagen), according to the manufacturer’s instructions. Single-cell RNA preparation was performed following the SMART-seq2 protocol^31^. Then complementary DNA (cDNA) was purified with 0.8 × Agencourt AMPure XP beads (Beckman Coulter, Brea, CA, USA), and 1 ng cDNA from each sample was used as the starting amount for library preparation. We used TruePrep^TM^ Mini DNA Sample Prep Kit (Vazyme Biotech, Nanjing City, China) to construct libraries, according to the instruction manual. All samples were sequenced on Illumina HiSeq 4000 sequencing system (Illumina, San Diego, CA, USA) with paired-end 100 bp × 2.

### Follow-up and tumor recurrence

Postoperative patient surveillance was performed, as previously described^32^. A diagnosis of intrahepatic recurrence (IHR) or EHM was based on computed tomography scans, magnetic resonance imaging, digital subtraction angiography, or positron emission tomography scans, with or without histological confirmation. Follow-up was terminated on October 30, 2017. TTR was defined as the interval between resection and the diagnosis of any type of recurrence^33^, with IHR or EHM defined as end points^34^. We defined recurrence within 2 years after surgical resection as early recurrence^35^. OS was defined as the interval between surgery and death or the last observation taken. For surviving patients, the data were censored at the last follow-up.

### Single-CTC preparation and low-pass whole-genome sequencing

Single CTC was isolated with a commercially available automated micromanipulation platform. Single-CTC whole-genome amplification and sequencing library was prepared using the SMARTer® PicoPLEX ® Gold Single-Cell DNA-Seq kit (Takara Biosystems), according to the manufacturer’s instructions. All 295 CTC were prepared to cDNA using SMART-seq2 approach. The libraries were quantified using Qubit dsDNA HS Assay kit with Qubit 2.0 Fluorometer (Thermo Fisher Scientific) and fragment analysis, using Agilent High-Sensitivity DNA Kit with Agilent Bioanalyzer 2100 (Agilent Technologies). A 119 single-cell cDNA with a peak of fragment between 1000 and 2000 bp, and concentration >0.3 ng/µl passed the cDNA quality control were sequenced on an Illumina Hiseq 4000 system (read lengths of 2 × 150 bp).

A strict two-step quality controls was used for single cell. The primary QC: we validate the amplification products by using electrophoresis and quantitative PCR (qPCR) assays. The secondary QC: we use Lorenz curve to confirm the coverage of sequencing data and evenness of sequencing reads LP-WGS of CTCs qPCR was performed for 12 randomly selected loci to check the genomic integrity of the single cell library. The libraries with 8 out of 12 loci amplified by qPCR with a reasonable Ct number were used for sequencing. The library was quality checked and sequenced on an Illumina HiSeq X Ten system (read lengths of 2 × 150 bp).

### Copy number variation analysis

The whole-genomics sequencing data was firstly mapped to hg19 using BWA 0.7.17, with the default arguments and then sorted by SAMtools 1.7. Duplications were marked by Picard 2.18.0. Afterward, CNVs were assessed by Ginkgo (http://qb.cshl.edu/ginkgo). Quality metrics data, including index of dispersion, Lorenz curve, and histogram of read count distribution, were calculated as a part of the Ginkgo analysis pipeline.

The inferred CNV result-based scRNA-seq data were performed by inferCNV R package (https://github.com/broadinstitute/inferCNV)^36^. WBCs from P9 were used as normal reference cells. For each cell, CNV inferred based on the average expression of large genes sets in each chromosomal region of the interrogating cell genome compared to normal cells. The following parameters were used for the inferCNV analysis: “denoise” mode, a value of 201 of “window_length”, and a value of 1 for “cutoff”. To reduce the noise of CNV calls a filtering gradient by applying a sigmoidal function was implemented to reduces intensities near the mean more than intensities more distant from the mean. The midpoint for the sigmoidal curve (logistic function) is set to 2 based on the “sd_amplifier”.

### Analysis of full-length scRNA-seq data

Paired-end 100 bp × 2 reads from Hiseq 4000 were filtered by using SOAPnuke1.5.0 with parameters “-l 5 -q 0.5 -n 0.1 -Q 2 -G −5 1.” The resulting FASTQ files were mapped to UCSC hg19 human genome and transcriptome (downloaded from http://genome.ucsc.edu/) using TopHat v2.0.12 (ref. ^37^), with parameters “-p 4 -g 1 -N 1 --read-gap-length 2 --read-edit-dist 2 --b2-very-sensitive --segment-length 24 --segment-mismatches 1 --mate-std-dev 20 --library-type fr-unstranded --fusion-search --fusion-min-dist 100000.” Gene expression levels were quantified by edgeR as fragments per kilobase of exon per million fragments mapped (FPKM). Transcript isoform abundances were estimated by RSEM v1.2.31 (ref. ^38^) in paired-end mode with default parameters.

We excluded cells with expressed genes (FPKM > 0) <3000 and unique mapping reads ≤ 1 M to improve accuracy. In total, 125 single cells (113 CTCs + 8 WBCs + 4 cell lines) and 14 bulks remained for further analysis. The number of mapped reads and detected genes are shown in the Supplementary information (Supplementary Data 3).

### Dimensionality reduction

Expression data from 113 CTCs, 4 cell lines, and 7 primary tumors were combined to a single dataset at first for an overview. FPKM of Genes expressed (FPKM > 1) in >10% of CTCs were transformed to log2 space. *t*-SNE was then preformed by R package “Rtsne” with parameters “pca = TRUE, perplexity = 30, theta = 0, dims = 2, and max_iter = 1000”. For dimensionality reduction of CTCs, the operational parameters were “pca = TRUE, perplexity = 8, theta = 0, dims = 2, and max_iter = 1000”.

### Unsupervised hierarchical clustering analysis

Unsupervised hierarchical clustering analysis of CTCs, cell lines, and primary tumors, as well as clustering within CTCs, were derived from Euclidean distance measurement of FPKM on log2 level.

### Differential expression analysis

Differential expression gene (DEG) analyses in pairs were performed with an edgeR algorithm^39^ or Wilcoxon signed-rank tests. DEGs detected with count per million (CPM) < 1 in more than two samples were filtered out. And only those with fold change > 1 (log2 level), *P* < 0.05, and false discovery rate (FDR) < 0.05 estimated by the Benjamini–Hochberg (BH) method were selected as initial DEGs. To determine genes that were significantly upregulated, we estimated the average expression in the corresponding group for initial DEGs. Only those with an average fold change of FPKM > 1 (log2 level) and expressed in at least 90% of samples in the group were selected as potential DEGs. Following Wilcoxon signed-rank tests, DEGs with statistical significance (*P* < 0.05) were selected as markers in the corresponding site.

### Heterogeneity

To compare heterogeneity among CTCs from different locations within and between subsets of specimens, we calculated means of correlation coefficients using the approach outlined in David et al.^40^. The top 2000 genes with the highest variance in log2 (FPKM + 1) values across all specimens were used for Pearson correlation coefficient calculation among CTCs. All patients with at least two CTCs at various sampling sites were included in the analysis of heterogeneity of various sites across patients, and P9 served as an example of individual heterogeneity exploration. A *P* value was assigned to each paired site using Student’s *t* tests.

### Cell cycle analysis

Itay et al.^41^ studied metastatic melanoma and defined a set of 42 G1/S and 54 G2/M genes known to function in replication and mitosis, respectively. Our previous study of scRNA-seq in HeLa cells also revealed a cell cycle signature. We therefore classified CTCs according to the unsupervised hierarchical clustering result based on those cycling genes. Log2 transformation was applied to gene expression. Then we recentered the data within the gene set by defining relative expression for gene *i* in sample *j* as *Ei*, *j* = log2(FPKM*i*, *j* + 1) − average[log2(FPKM*i*, 1 + 1), log2(FPKM*i*, 2 + 1), … log2(FPKM*i*, *n* + 1)]. Averaging the relative expression *Ei*, *j* of G1/S and G2/M gene sets for each cell revealed their cell cycle status.

### Time-series analysis

To investigate the transcriptional dynamics of CTCs during blood circulation, we performed pseudotemporal analysis on CTCs captured from different vascular sites using the R package Monocle^42^. Genes expressed in at least 10% cells with FPKM > 1 were taken as inputs. The pseudotemporal path number was set 2.

### Gene set enrichment

DEGs were used to perform enrichment analysis using GSEA^43^ on HALLMARK (h.all.v6.1.symbols.gmt), REACTOME (c2.cp.reactome.v6.1.symbols.gmt), GO(BP), and KEGG gene sets in the GSEA software built locally. FDR was estimated by BH with a threshold of 0.05.

### Cell line experiments

Three HCC cell lines (Huh7, Hep3B, and Hepa1–6) were purchased from the Shanghai Cell Bank, Chinese Academy of Sciences. MHCC97H (highly metastatic human HCC cell lines) and MHCC97L (low metastatic human HCC cell lins) were established at our institute. The cell line was characterized by the cell bank based on cell morphology, post-freeze viability, isoenzyme analysis, DNA fingerprinting analysis, mycoplasma contamination testing, and bacterial and fungal contamination. All cell lines were cultured in Dulbecco’s modified Eagle’s medium (DMEM) containing 10% fetal bovine serum (FBS) supplemented with 100 IU/ml penicillin and 100 µg/ml streptomycin, and incubated at 37 °C under a humidified atmosphere with 5% CO_2_. All GIBCO cell culture reagents were obtained from Invitrogen (Carlsbad, CA, USA).

### shRNA-mediated CCL5 and MAX knockdown

We generated lentiviruses encoding nonsilencing-shRNA named vector-shRNA (control) and CCL5-specific or MAX-specific shRNA (knockdown). For lentiviral transduction, Hepa1–6 cells were cultured in six-well plates to 70% confluence, and infected with vector-shRNA or CCL5-specific or MAX-specific shRNA lentivirus using lipofectamine.

### siRNA-mediated MAX knockdown

Transfection of siRNA was done using Lipofectamine 2000 (Invitrogen), according to the manufacturer’s instructions. The target siRNA sequences used were as follows:

siMAX#1: 5′-TCAATCTGCGGCTGACAAA-3′

siMAX#2: 5′-GGGCCCAAATCCTAGACAA-3′

### Flow cytometry analysis of Tregs

Flow cytometry analysis was conducted with an Aria II flow cytometer (BD Biosciences). For surface staining, peripheral blood mononuclear cell suspensions were stained on ice using predetermined optimal concentrations of each antibody for 30 min, and fixed using fixation buffer (BD Bioscience, San Diego, CA). Tregs identified with CD4^+^CD25^+^CD127^−^ expression were stained with human Treg Cocktail (BD Biosciences). Data analysis was performed using FlowJo software (FlowJo, Ashland, OR, US).

### Measurement of cytokines by cytometric bead array

We used a BD^TM^ CBA assay (BD Biosciences) to measure TGF-β1, IL-10, IL-35, VEGF, and TNF-α. The assay procedure was performed according to the manufacturer’s instructions. Briefly, six bead populations with distinct fluorescence intensities were coated with capture antibodies specific for each molecule. The six bead populations were mixed together and were resolved in the FL3 channel of a BD fluorescence-activated cell sorter. The cytokine-capture beads were mixed with the PE-conjugated detection antibodies, and then incubated with recombinant standards or test samples to form sandwich complexes. Following acquisition of sample data using the flow cytometer, the sample results were tabulated and graphed using the BD CBA Analysis Software. Cytokines in each sample were measured in triplicate in the experiments, and the data are presented as mean values ± SD.

### Tregs migration assay

An HCC cell line-peripheral Treg coculture system was established to investigate the association between migratory abilities of Tregs and HCC cell-derived CCL5. Cell migration was assessed as described^44^, using CD4^+^CD25^+^ Tregs isolated (130-091-301; Miltenyi Biotec) from the peripheral blood of HCC patients. Tumor cells were plated in the lower chamber, and Tregs were plated in the upper chamber. The culture medium used in coculture system was DMEM (GIBCO, Invitrogen) without FBS. Antibodies against CCL5 (Cat# MAB678, R&D systems) and control IgG (Cat# 1-001-A, R&D systems) were obtained from R&D Systems. After 48 h, the migrated Tregs that crossed the inserts were stained with crystal violet (0.005%, Sigma) and counted as cells per field under 20 × phase-contrast microscopy.

### Chromatin immunoprecipitation

DNA and associated proteins on chromatin in cultured cells were crosslinked by 1% formaldehyde for 15 min at 37 °C. Cells were then scraped and collected in cellular lysis buffer (5 mM Pipes, 85 mM KCl, 0.5% NP-40, and protease inhibitors). Cytoplasmic lysates were discarded and nuclear components were resuspended in nuclear lysis buffer (50 mM Tris pH 8, 10 mM EDTA pH 8, 0.2% SDS, and protease inhibitors), and sonicated for 10 min (Covaris). Approximately 4 mg of MAX antibody (Cat # ab53570, diluted 1:800, Abcam) or control IgG (Cat # ab6715, diluted 1:1000, Abcam) were incubated with 25 ml of protein G magnetic beads for 6 h at 4 °C, and then incubated with 100 mg of cleared chromatin overnight at 4 °C. After three washes, immunoprecipitated material was eluted at 55 °C for 1 h with 10 µg/ml proteinase K, and then decrosslinked at 65 °C for 4 h. The primer sequences used for ChIP-qPCR are listed as follows:

**Table:** 

Gene	TSS distance	Primer
*GAPDH*	−400	Foward primer: CGCCTCTCAGCCTTTGAAAGAAA
		Reverse primer: TTGGATGAAACAGGAGGACTTTG
*RPL30*	−150	Foward primer: TTGAGCTGGACGCAACAG
		Reverse primer: GCGGCTGCTCATACCTTT
*CCL5*	−800	Foward primer: AGGACAGTGGAATAGTGGCTGG
		Reverse primer: ACTTGTTGAGAAGCAGAGGGAGAG

### Dual-luciferase reporter gene assay

Dual-luciferase reporter gene assays were performed using the Dual-Luciferase Reporter Assay System (Promega, Madison, WI, USA). MHCC97H cells were transiently transfected with −1000/+50 MAX promoter pGL3 basic reporter construct and pRLTK Renilla luciferase plasmid (Promega) as a normalization control, using Lipofectamine 2000 (Invitrogen).

### Western blot

Cell lysates and supernatants were resolved by electrophoresis, transferred to a polyvinylidene fluoride membrane, and probed with antibodies against GAPDH (Cat# sc-47724, dilated 1:600, Santa Cruz Biotechnology), CCL5 (Cat# 2988 S, diluted 1:1000, Cell Signaling Technology), or MAX (Cat# 4739 S, diluted 1:800, Cell Signaling Technology).

### cDNA microarray

cDNA expression profiling was performed using total RNA with the GeneChip Human Genome U133 Plus 2.0 Array (Affymetrix, Santa Clara, CA, USA), according to the manufacturer’s instructions and a previous report^45^.

### Immunohistochemistry assay

Tumoral and peritumoral specimens from ten patients paired with CTC scRNA-seq data were formalin-fixed, paraffin-embedded, sectioned, and stained with hematoxylin and eosin for histopathological evaluation at the pathology department of Zhongshan Hospital. IHC studies employed 5-mm sections of formalin-fixed, paraffin-embedded tissue. All were stained on the Leica Bond III automated platform using the Leica Refine detection kit (Leica Microsystems, Wetzlar, Germany). Sections were deparaffinized, and heat-induced epitope retrieval was performed on the unit using EDTA for 20 min at 90 °C. Sections were incubated for 30 min with primary antibody FoxP3 (Cat# ab20034, diluted 1:250, Abcam) and CCL5 (Cat# AF-278-NA, diluted 1:250, R&D Systems). The EnVision G/2 Double stain system (DAKO) was used for dual-color antigen staining. All tissues were counterstained with hematoxylin. Isotype controls used were rabbit immunoglobulin fraction and mouse IgG1 from DAKO. Whole tissue sections were imaged with a LeicaSCN400 histology scanner. All sections were evaluated by two pathologists without knowing patient clinical characteristics and outcomes.

### Multiplex immunofluorescence staining assay

Multiplex staining of was performed using TSA 7-color kit (D110071-50T, Yuanxibio), according to manufacturer’s instruction. Primary antibodies included two panels: the first panel was CK8/18 (Cat# BX50145, diluted 1:800, Biolynx), FoxP3 (Cat# MAB8214, diluted 1:100, R&D systems), CCL5 (Cat# AF-278-NA, diluted 1:250, R&D systems), and CD8 (Cat# BX50036-C3, diluted 1:300, Biolynx); the second panel was CK8/18, FoxP3, CCL5, and CCR5 (Cat# MAB182-100, diluted 1:100, R&D systems); the third panel was CK8/18, CCL5, FoxP3, CD3 (Cat# BX50022, diluted 1:100 Biolynx), and CD45RO (Cat# GM074202, ready-to-use, Gene Tech). Primary antibodies were sequentially applied, followed by horseradish peroxidase-conjugated secondary antibody incubation (1:1, Cat# DS9800, Lecia Biosystems; 1:1 Cat# A10011-6/A10012-6, WiSee Biotechnology), and tyramide signal amplification (M-D110051, WiSee Biotechnology). The slides were microwave heat-treated after each TSA operation. Nuclei were stained with DAPI (D1306, ThermoFisher) after all the antigens above being labeled. The stained slides were scanned to obtain multispectral images using the Pannoramic MIDI imaging system (3D HISTECH). The peritumoral tissue of five patients from CTC scRNA-seq cohort were evaluated by multiplex IF assay. Five randomly selected peritumoral microvasculature from each patient were counted for the number of target cells by HALO Software (Indica Labs).

### Animal studies

All research involving animals complied with protocols approved by the Zhongshan Hospital Animal Care and Use Committee. Six- to eight-week-old male C57BL/6J mice were treated under the following conditions. For assessment of CTC circulatory clearance rate, mice were injected with 5 × 10^6^ control or shCCL5 Hepa1–6 cells via tail vein. Antimouse CD25 antibody (Cat# BE0012, clone PC61, Rat IgG1, BioXcell) or control IgG (Cat# BE0088, clone HRPN, Rat IgG1 BioXcell) was injected intraperitoneally at −1 and 0 days before tumor cell injection via the tail vein. Murine blood samples were collected by intracardiac puncture at each time point of the experiment and processed by RBC lysis, and then stained with PE-labeled antimouse pan-CK antibody and Alexa-488 antimouse CD45 antibody. Blood samples were then subjected to IF analysis. For evaluation of CTC metastatic tumorigenesis, 5 × 10^6^ control, shCCL5, or shMAX Hepa1–6 cells were injected intravenously or intrasplenically. Antimouse CD25 antibody or control IgG was injected intraperitoneally at −1, 3, 10, 18, and 27 days of tumor cell injection. For subcutaneous tumorigenesis, 1 × 10^6^ control, shCCL5, or shMAX Hepa1–6 cells were inoculated subcutaneously.

The liver metastases were processed with formalin fixation, paraffin embedded, and sectioned to 5 μm in thickness. IHC was performed with a standard protocol. Antibodies used are FOXP3 (Cat# 2A11G9, diluted 1:200, Santa Cruz), CD8 (Cat# 6A242, diluted 1:200, Santa Cruz), and granzyme B (Cat# sc8022, diluted 1:250, Santa Cruz). Tregs and GrB^+^ cells were counted under microscopy (20× objective lens). Five independent microscopic field, representing the densest lymphocytic infiltrates, were selected for one liver metastatic tumor each mouse to ensure representativeness and homogeneity. The results were expressed as the mean (±SD) number cells for one microscopic field. GrB^+^ cells with a sparsely granulated pattern were evaluated as activated CTLs.

### Imaging of lung metastases in mice

For in vivo imaging with micro-computed tomography (microCT; Latheta LCT-200, Hitachi-Aloka, Japan), mice were anesthetized with oxygen (0.5 l/min) and isoflurane (1–1.5 vol %). MicroCT was performed at 50 kV, with an anode current of 0.5 mA. Scans were completed over 360° of rotation of the x-ray tube. The resolution of the scanning was 96 µm pixel. No contrast agent was applied to image lung metastases. Respiratory triggering was used to reduce movement artifact from animal breathing and internal organ movement, and images were captured after the peak of expiration and beginning of the peak of inspiration, when the period without movement was the longest one and was usually matching with 500 ms time, as was the time of the shutter speed.

### Statistical analysis

Statistical analyses were performed using SPSS version 19.0 for windows (IBM, Armonk, NY, USA). All experiments had at least one additional independent repeat with similar results. Data were presented as means and standard deviations or medians and ranges. The unpaired two-tailed Student’s *t* test was used to examine differences between two groups. Kaplan–Meier survival curves and a log-rank testing were performed to estimate the prognostic significance. Univariate and multivariate analyses were based on the Cox proportional hazards regression model. The median numbers of CTCs and circulating Treg/CD4^+^ T cells (%) were used as cutoff values in Kaplan–Meier survival curves, univariate, and multivariate analysis. A two-sided *P* < 0.05 was considered statistically significant.

### Reporting summary

Further information on research design is available in the Nature Research Reporting Summary linked to this article.

## Supplementary information

Supplementary Information

Description of Additional Supplementary Files

Supplementary Data 1

Supplementary Data 2

Supplementary Data 3

Supplementary Data 4

Supplementary Data 5

Supplementary Data 6

Reporting Summary

## Data Availability

Data generated for this study is available in the European Genome-phenome Archive (EGA, https://ega-archive.org/studies/EGAS00001005204) under restricted access. The data can be accessed by contacting the author named on the EGA website. The data are also available through the CNGB Nucleotide Sequence Archive (GNSA, https://db.cngb.org/cnsa/, CNP0000095). All remaining data are available within the article or Supplementary Information.

## References

[1] Zhou, J. et al. Guidelines for diagnosis and treatment of primary liver cancer in China (2017 Edition). *Liver Cancer* **7**, 235–260 (2018). 10.1159/000488035 PMC6167671 30319983

[2] Poon RT (2002). Tumor microvessel density as a predictor of recurrence after resection of hepatocellular carcinoma: a prospective study. J. Clin. Oncol. Off. J. Am. Soc. Clin. Oncol..

[3] Mann J, Reeves HL, Feldstein AE (2018). Liquid biopsy for liver diseases. Gut.

[4] Senft D, Ronai ZA (2016). Adaptive stress responses during tumor metastasis and dormancy. Trends Cancer.

[5] Miyamoto DT (2015). RNA-Seq of single prostate CTCs implicates noncanonical Wnt signaling in antiandrogen resistance. Science.

[6] Yu M (2013). Circulating breast tumor cells exhibit dynamic changes in epithelial and mesenchymal composition. Science.

[7] Sun YF (2018). Circulating tumor cells from different vascular sites exhibit spatial heterogeneity in epithelial and mesenchymal composition and distinct clinical significance in hepatocellular carcinoma. Clin. Cancer Res..

[8] Guo W (2014). Clinical significance of EpCAM mRNA-positive circulating tumor cells in hepatocellular carcinoma by an optimized negative enrichment and qRT-PCR-based platform. Clin. Cancer Res..

[9] Ogle LF (2016). Imagestream detection and characterisation of circulating tumour cells - a liquid biopsy for hepatocellular carcinoma?. J. Hepatol..

[10] Motohashi H, Katsuoka F, Shavit JA, Engel JD, Yamamoto M (2000). Positive or negative MARE-dependent transcriptional regulation is determined by the abundance of small Maf proteins. Cell.

[11] Labelle M, Begum S, Hynes RO (2014). Platelets guide the formation of early metastatic niches. Proc. Natl Acad. Sci. USA.

[12] Northcott PA (2014). Enhancer hijacking activates GFI1 family oncogenes in medulloblastoma. Nature.

[13] Strilic B, Offermanns S (2017). Intravascular survival and extravasation of tumor cells. Cancer cell.

[14] Macosko EZ (2015). Highly parallel genome-wide expression profiling of individual cells using nanoliter droplets. Cell.

[15] Ting DT (2014). Single-cell RNA sequencing identifies extracellular matrix gene expression by pancreatic circulating tumor cells. Cell Rep..

[16] Mohme M, Riethdorf S, Pantel K (2017). Circulating and disseminated tumour cells - mechanisms of immune surveillance and escape. Nat. Rev. Clin. Oncol..

[17] Serrels A (2015). Nuclear FAK controls chemokine transcription, Tregs, and evasion of anti-tumor immunity. Cell.

[18] Fishilevich, S. et al. GeneHancer: genome-wide integration of enhancers and target genes in GeneCards. *Database* **2017**, bax028 (2017). 10.1093/database/bax028 PMC5467550 28605766

[19] Wolf AM (2003). Increase of regulatory T cells in the peripheral blood of cancer patients. Clin. Cancer Res..

[20] Shi J (2016). Circulating tumour cells as biomarkers for evaluating cryosurgery on unresectable hepatocellular carcinoma. Oncol. Rep..

[21] Li J (2016). pERK/pAkt phenotyping in circulating tumor cells as a biomarker for sorafenib efficacy in patients with advanced hepatocellular carcinoma. Oncotarget.

[22] Gu X (2016). Increased programmed death ligand-1 expression predicts poor prognosis in hepatocellular carcinoma patients. Onco Targets Ther..

[23] Qi LN (2018). Circulating tumor cells undergoing EMT provide a metric for diagnosis and prognosis of patients with hepatocellular carcinoma. Cancer Res..

[24] Keller L, Pantel K (2019). Unravelling tumour heterogeneity by single-cell profiling of circulating tumour cells. Nat. Rev. Cancer.

[25] Plaks V, Koopman CD, Werb Z (2013). Cancer. Circulating tumor cells. Science.

[26] Miyamoto DT (2012). Androgen receptor signaling in circulating tumor cells as a marker of hormonally responsive prostate cancer. Cancer Discov..

[27] Lee HJ (2017). Fluid shear stress activates YAP1 to promote cancer cell motility. Nat. Commun..

[28] Ghajar CM (2015). Metastasis prevention by targeting the dormant niche. Nat. Rev. Cancer.

[29] Kurose K (2015). Phase Ia study of FoxP3+ CD4 treg depletion by infusion of a humanized anti-CCR4 antibody, KW-0761, in cancer patients. Clin. Cancer Res..

[30] Sun YF (2013). Circulating stem cell-like epithelial cell adhesion molecule-positive tumor cells indicate poor prognosis of hepatocellular carcinoma after curative resection. Hepatology.

[31] Picelli S (2014). Full-length RNA-seq from single cells using Smart-seq2. Nat. Protoc..

[32] Yang XR (2008). Cytokeratin 10 and cytokeratin 19: predictive markers for poor prognosis in hepatocellular carcinoma patients after curative resection. Clin. Cancer Res. Off. J. Am. Assoc. Cancer Res..

[33] Llovet JM (2008). Design and endpoints of clinical trials in hepatocellular carcinoma. J. Natl Cancer Inst..

[34] Yang XR (2010). High expression levels of putative hepatic stem/progenitor cell biomarkers related to tumour angiogenesis and poor prognosis of hepatocellular carcinoma. Gut.

[35] Shah SA (2006). Factors associated with early recurrence after resection for hepatocellular carcinoma and outcomes. J. Am. Coll. Surg..

[36] Patel AP (2014). Single-cell RNA-seq highlights intratumoral heterogeneity in primary glioblastoma. Science.

[37] Trapnell C (2012). Differential gene and transcript expression analysis of RNA-seq experiments with TopHat and Cufflinks. Nat. Protoc..

[38] Islam S (2011). Characterization of the single-cell transcriptional landscape by highly multiplex RNA-seq. Genome Res..

[39] Robinson MD, McCarthy DJ, Smyth GK (2010). edgeR: a Bioconductor package for differential expression analysis of digital gene expression data. Bioinformatics.

[40] Miyamoto DT (2015). RNA-Seq of single prostate CTCs implicates noncanonical Wnt signaling in antiandrogen resistance. Science.

[41] Tirosh I (2016). Dissecting the multicellular ecosystem of metastatic melanoma by single-cell RNA-seq. Science.

[42] Trapnell C (2014). The dynamics and regulators of cell fate decisions are revealed by pseudotemporal ordering of single cells. Nat. Biotechnol..

[43] Subramanian A (2005). Gene set enrichment analysis: a knowledge-based approach for interpreting genome-wide expression profiles. Proc. Natl Acad. Sci. USA.

[44] Curiel TJ (2004). Specific recru itment of regulatory T cells in ovarian carcinoma fosters immune privilege and predicts reduced survival. Nat. Med..

[45] Lee TK (2011). CD24(+) liver tumor-initiating cells drive self-renewal and tumor initiation through STAT3-mediated NANOG regulation. Cell Stem Cell.

