# Supplementary material for: Dissecting spatial heterogeneity and the immune-evasion mechanism of CTCs by single-cell RNA-seq in hepatocellular carcinoma
Source: Nat Commun. 2021 Jul 2;12:4091. doi: 10.1038/s41467-021-24386-0 (PMC8253833; doi:10.1038/s41467-021-24386-0)
Supplement: Supplementary file 1 — Supplementary Information [file 41467_2021_24386_MOESM1_ESM.pdf]

Supplementary Figure 1

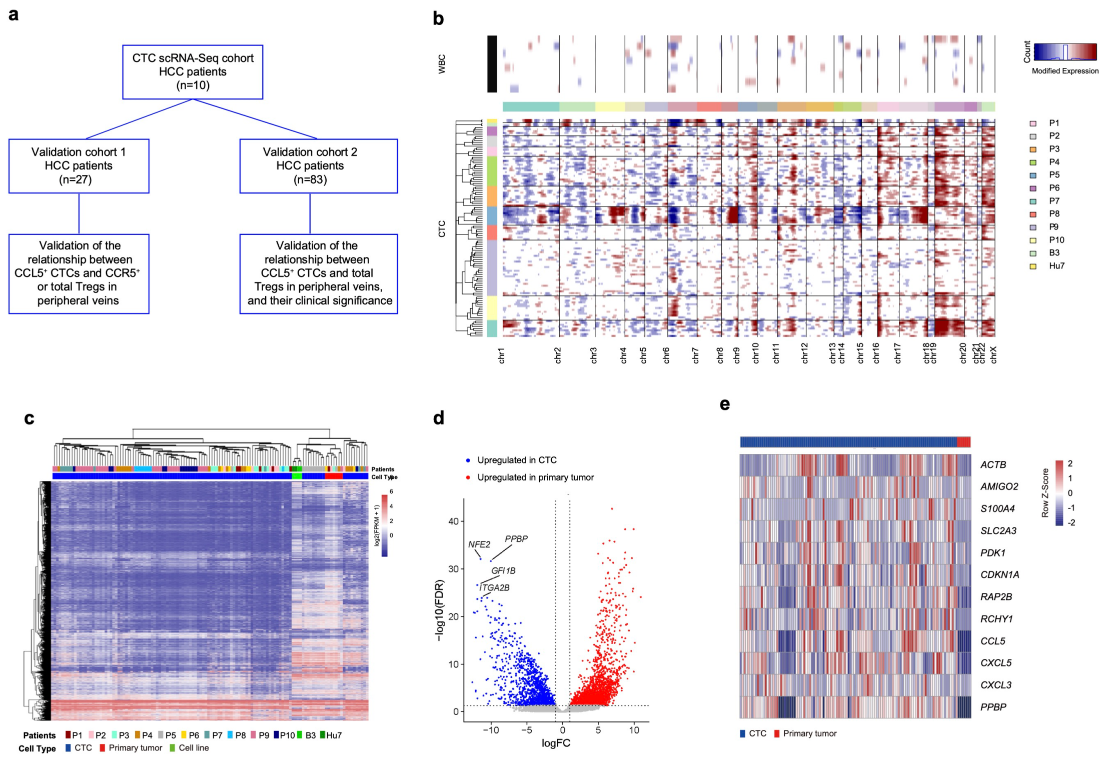

Supplementary Figure 1. The Overview of Patients Enrolled and Differential Expression Analysis of CTCs and Primary Tumors, Related to Figure 1

a An overview of patients enrolled in the current study. CTC, circulating tumor cells; HCC, hepatocellular carcinoma; scRNA-seq, single-cell RNA sequencing; Treg, regulatory T cell. **b** Chromosomal landscape of inferred large-scale CNVs of 113 CTCs from 10 HCC patients and two HCC cell lines including Hep3B and Huh7. **c** Heat map showing gene expression patterns in CTCs, primary tumors, and cell lines. **d** Genes differentially expressed in CTCs and primary tumors. (FDR < 0.05, fold change > 2). **e** Heat map showing that CTCs exhibit upregulation of genes implicated in energy metabolism reprogramming, DNA repairing and chemokine signaling pathways compared with primary tumors.

Supplementary Figure 2

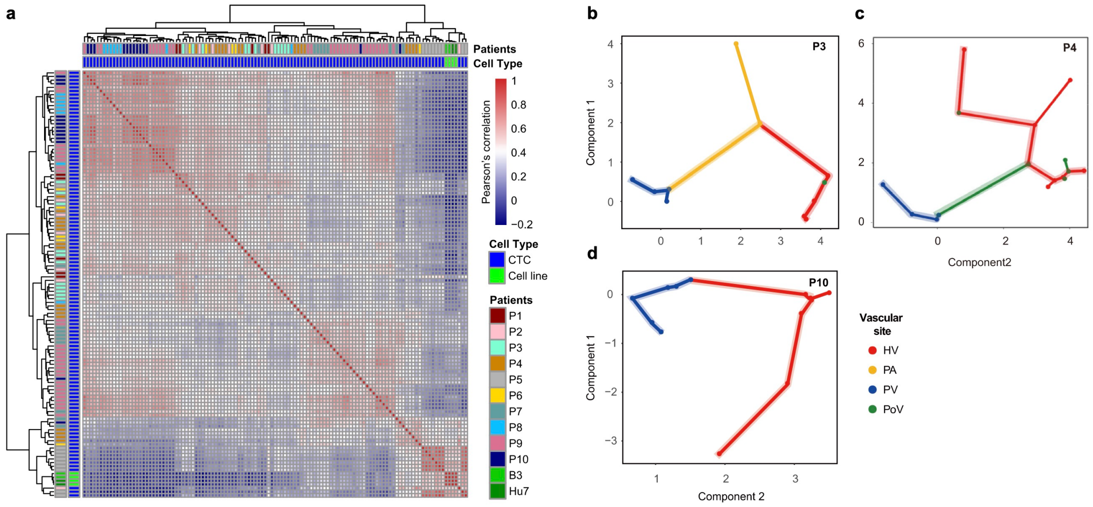

Supplementary Figure 2. The Inter- and Intra-patient Transcriptional Heterogeneity in CTCs, Related to Figure 2

a Heat map displaying the correlation of global expression profile between CTCs and HCC cell lines. **b-d** Pseudotemporal ordering of CTCs in patients P3 (b), P4 (c) and P10 (d), who has more than three cells collected in at least two sampling sites. HV, hepatic vein; PA, peripheral artery; PoV, portal vein; PV, peripheral vein.

Supplementary Figure 3

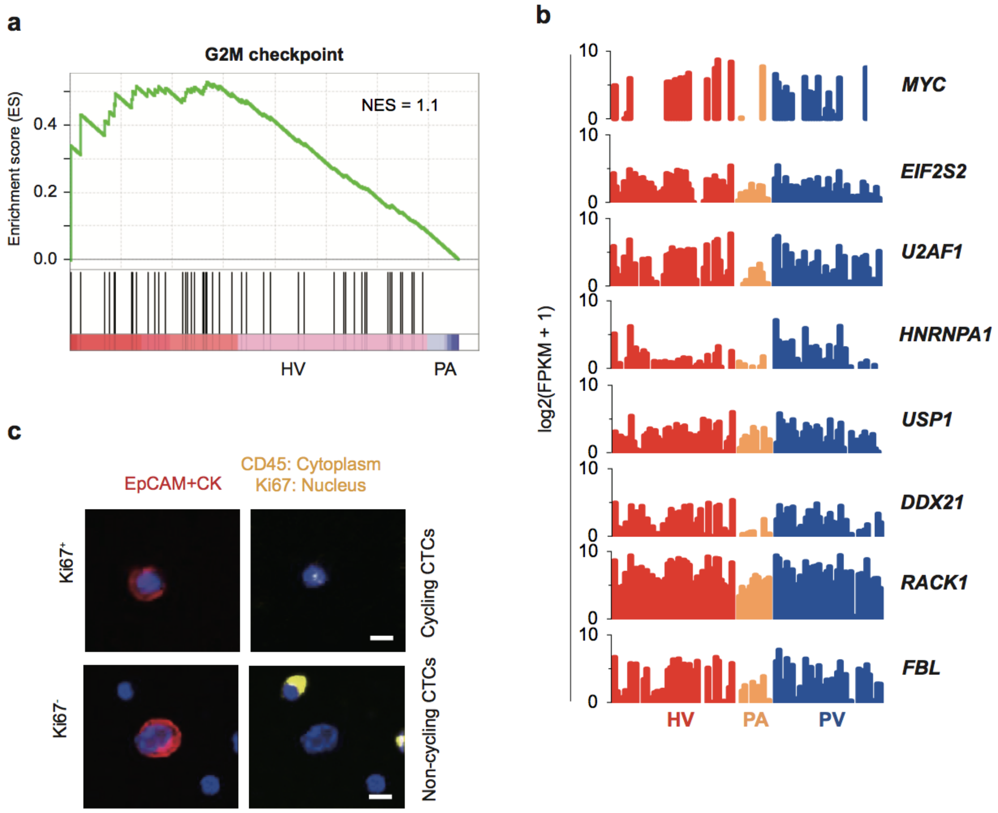

Supplementary Figure 3. Transcriptional Dynamics Associated with Cycling Cells in CTCs Between Neighboring Vascular Sites, Related to Figure 3

a GSEA enrichment chart of G2M checkpoint genes in HV and PA CTCs. **b** Histogram exhibiting the transient downregulation and subsequent upregulation of genes related to MYC targets in CTCs during their circulation process (HV→PA→PV). **c** Immunofluorescence images of Ki67 expression in cycling and non-cycling CTCs. Scale bar is 10 µm.

Supplementary Figure 4

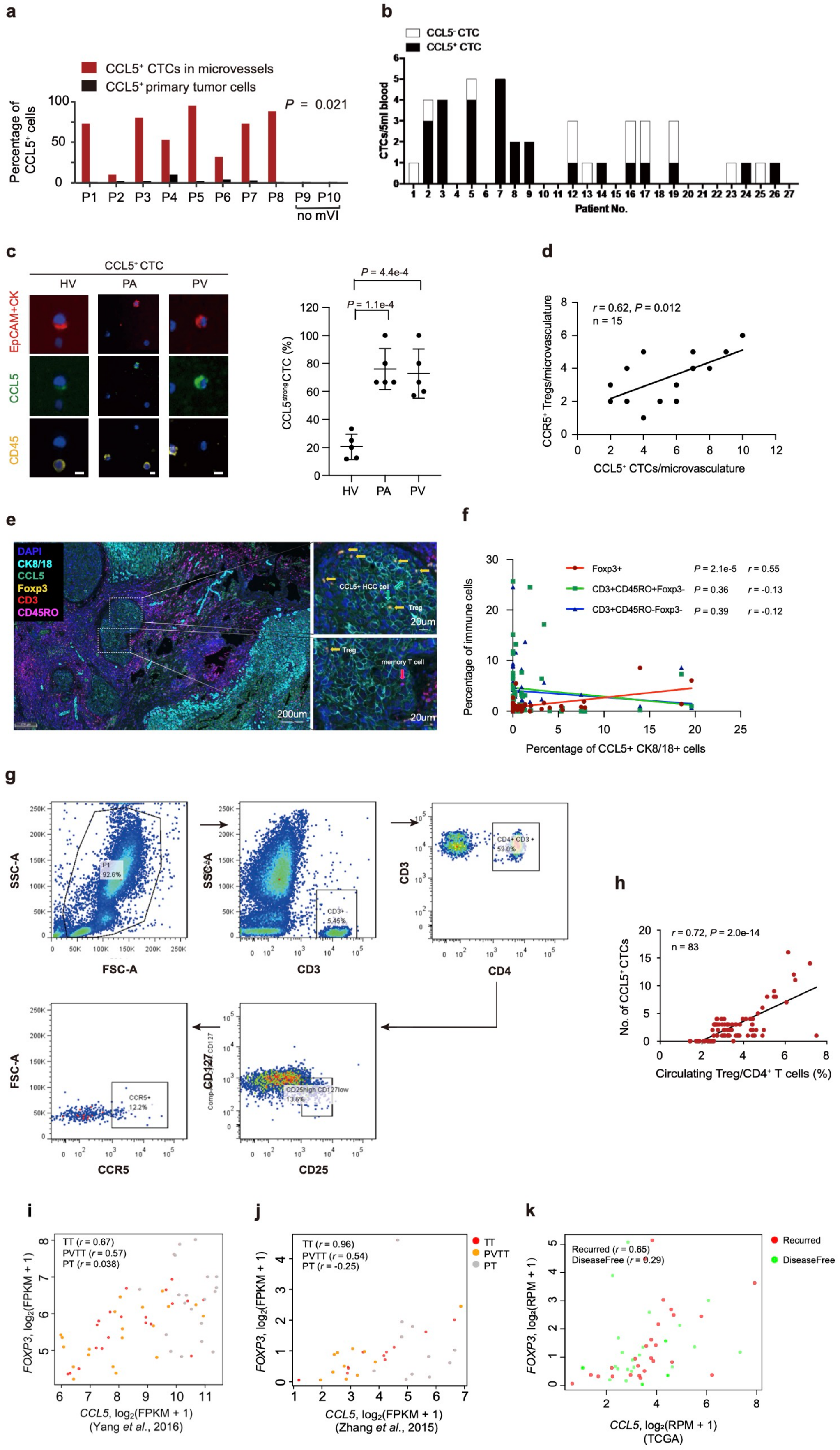

Supplementary Figure 4. Comparison of Immune Escape-Related Gene Expression in CTCs and Primary Tumors and The Correlation of $CCL5^+$ CTCs with Tregs, Related to Figure 4

a Histogram showing the percentage of $CCL5^+$ tumor cells in primary tumor and peritumoral microvasculature, respectively (lower). mVI, microvascular invasion. **b** Histogram showing the number of $CCL5^+$ and $CCL5^-$ CTCs in HCC patients from Cohort 1 (n = 27). **c** Immunofluorescence image showing CCL5 expression in CTCs from different vascular sites (HV, PA, PV). Scale bars, 10 μm. Scatterplot showing that the proportion of $CCL5^{strong}$ CTCs were significantly higher in PA and PV than that in HV (right panel). **d** Scatterplot showing a positive correlation between the number of $CCL5^+$ CTCs and $CCR5^+$ Tregs in peritumoral microvasculature. **e** Multiplex immunofluorescence images displaying the spatial relationship among $CCL5^+$ tumor cells, Tregs and $CD45RO^+$ T cells in peritumoral microvasculature. Scale bars represent 20 μm and 200 μm, respectively. **f** Scatterplot showing correlations between the number of $CCL5^+$ CTCs and Tregs, $CCL5^+$ CTCs and $CD45RO^+$ T cells, $CCL5^+$ CTCs and $CD45RO^-$ T cells in peritumoral microvasculature. **g** FACS result showing $CCR5^+$ Tregs in HCC patients. **h** Scatterplot showing a positive correlation between the number of $CCL5^+$ CTCs and total Tregs in $CD4^+$ T cells in peripheral blood from 83 HCC patients. **i-j** Scatterplots showing the correlation of *CCL5* and *FOXP3* expression in tumor tissue, PVTT and peritumoral tissue in published data sets from Yang et al. (i), Zhang et al. (j). PT, peritumoral tissue; PVTT, portal vein tumor thrombosis; TT, tumoral tissue. **k** Scatterplots showing *CCL5* and *FOXP3* expression in TCGA. Samples with tumor relapsed after initial treatment are in red, and those with disease-free records are in green. Comparisons were calculated by two-tailed Student's t-test (**c**). Data are mean±SD. Each dot represents data from an individual patient. Pearson correlation test was employed (**d, f**).

Supplementary Figure 5

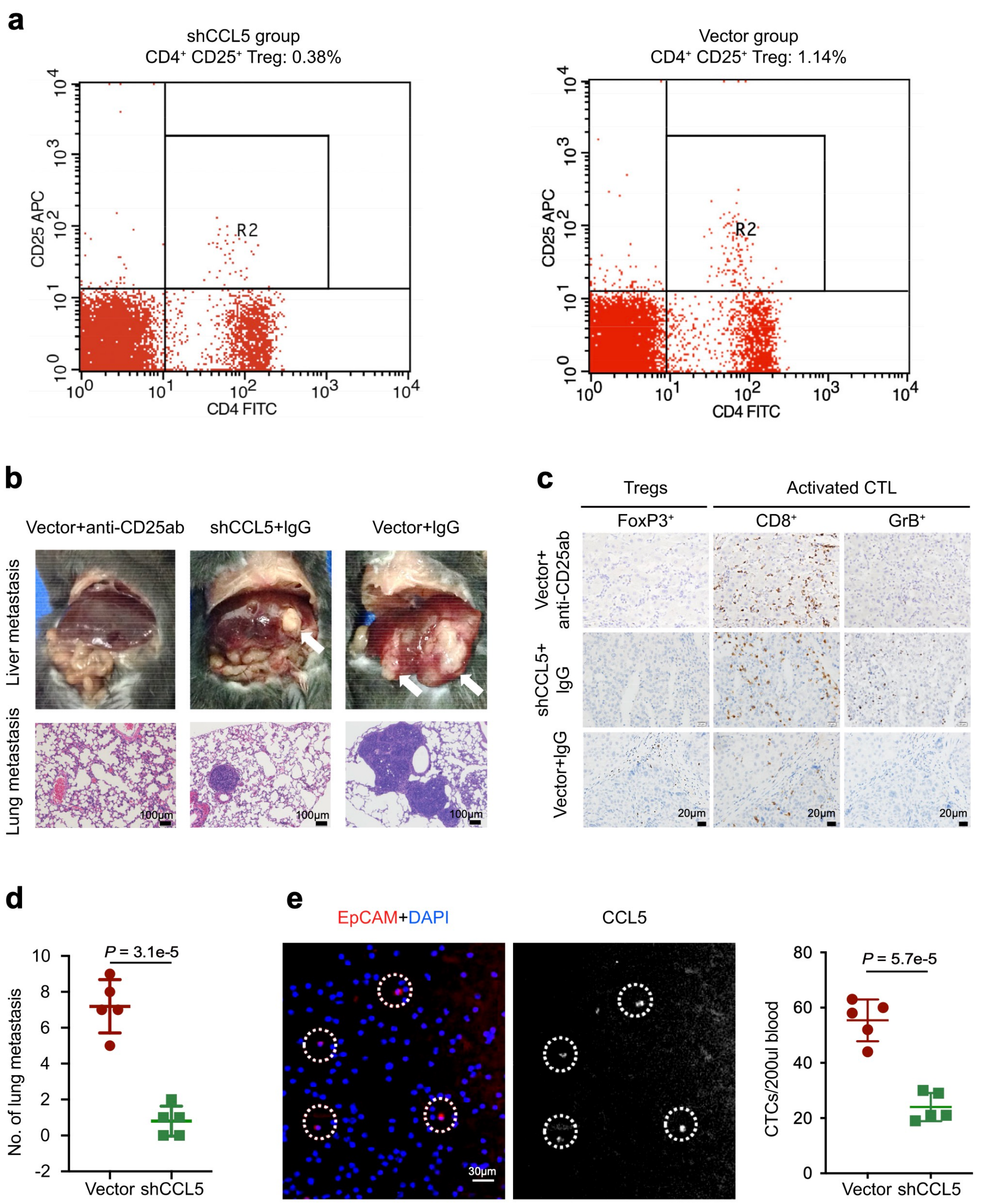

Supplementary Figure 5. The Impact of CCL5 on Tumor Growth and Metastasis Associated with Treg Recruitment, Related to Figure 5

a FACS results showing proportion of circulating Tregs in C57BL/6J mice of shCCL5 and vector control group. **b** Representative images showing the formation of liver and lung metastases in C57BL/6J mice with each of the indicated treatment conditions. White arrows, liver metastases. Scale bars represent 100μm. **c** Example immunohistochemistry image showing the association of tumor-infiltrated Tregs and activated CTLs in intrahepatic metastases from C57BL/6J mice treated with the indicated conditions. CTL, cytotoxic T lymphocyte. Scale bars represent 20 μm. **d** Scatter plots showing the numbers of lung metastatic lesions in orthotopic liver cancer model of C57BL/6J mice inoculated with shCCL5 or vector control Hepa1-6 cells. **e** Representative immunofluorescence images of $CCL5^{+}$ CTCs isolated from mice

blood and scatter plots showing CTC numbers per 200μl blood in orthotopic liver cancer model of C57BL/6J mice inoculated with shCCL5 or vector control Hepa1-6 cells. Scale bars represent 30 μm. Comparisons were calculated by two-tailed Student's t-test. Data are mean±SD of five biological replicates (**d, e**) and are representative of two independent experiments.

Supplementary Figure 6

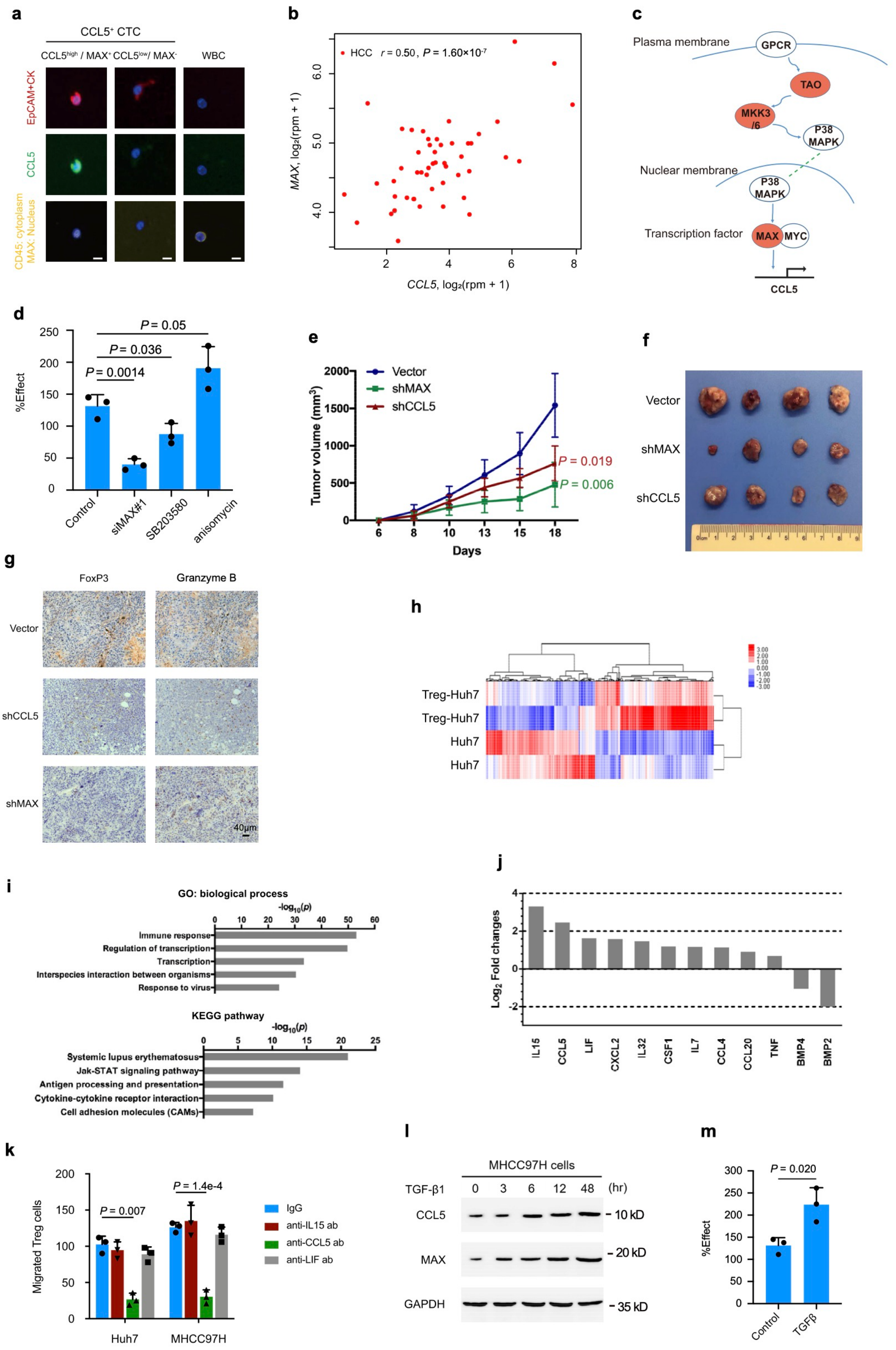

Supplementary Figure 6. The induction of CCL5 expression is mediated by transcriptional factor MAX and the Treg-derived TGF-β1, Related to Figure 6

a Detection of MAX and CCL5 expression in CTC by immunofluorescence assay. Scale bars represent 10 µm. **b** Scatterplots showing a significant correlation of *CCL5* and *MAX* expression in HCC tumoral tissue in published data sets from TCGA. Pearson correlation test was employed. **c** p38-MAPK signaling pathway. Components of pathway enriched in CTCs based on GSEA analysis are highlighted in red. **d** Relative luciferase activity of CCL5 promoter treated with siMAX#1, p38 inhibitor (SB203580) and p38 activator (anisomycin). **e** Tumor growth measured by volume after subcutaneous injection with 5×10^6 Hepa1-6 murine HCC cells with three different conditions (vector Hepa1-6 cells, CCL5-knockdown Hepa1-6 cells and MAX-knockdown Hepa1-6 cells) in immune-competent C57BL/6J mice (n = 4 mice per group). **f** Pictures showing the tumor size treated with three different condition as describe in figure e (n = 4 mice per group). **g** Example immunohistochemistry image showing the association of tumor-infiltrated Tregs and activated CTLs in lung metastases from C57BL/6J mice treated with the indicated conditions. Scale bars represent 40 µm. **h** Heat map displaying differentially expressed genes between Huh7 cells cocultured with or without freshly isolated peripheral Tregs of HCC patients. **i** GO and KEGG analysis for differential gene set. **j** Twelve differentially expressed chemotactic cytokines between Huh7 cells co-cultured with circulating Tregs from HCC patients and untreated Huh7 cells. **k** The numbers of migrated Tregs co-cultured with supernatant medium of Huh7 or MHCC97H cells treated with anti-human IL-15, CCL5 or LIF neutralizing antibody or IgG. **l** Time course of TGF-β1-induced expression of CCL5, MAX in MHCC97H cell line. **m** Relative luciferase activity of CCL5 promoter treated with TGF-β1. Comparisons were calculated by two-tailed Student's t-test (d, e, k, m). Data are mean±SD of three biological (d, k, and m) and four biological (e) replicates and are representative of two independent experiments.

Supplementary Figure 7

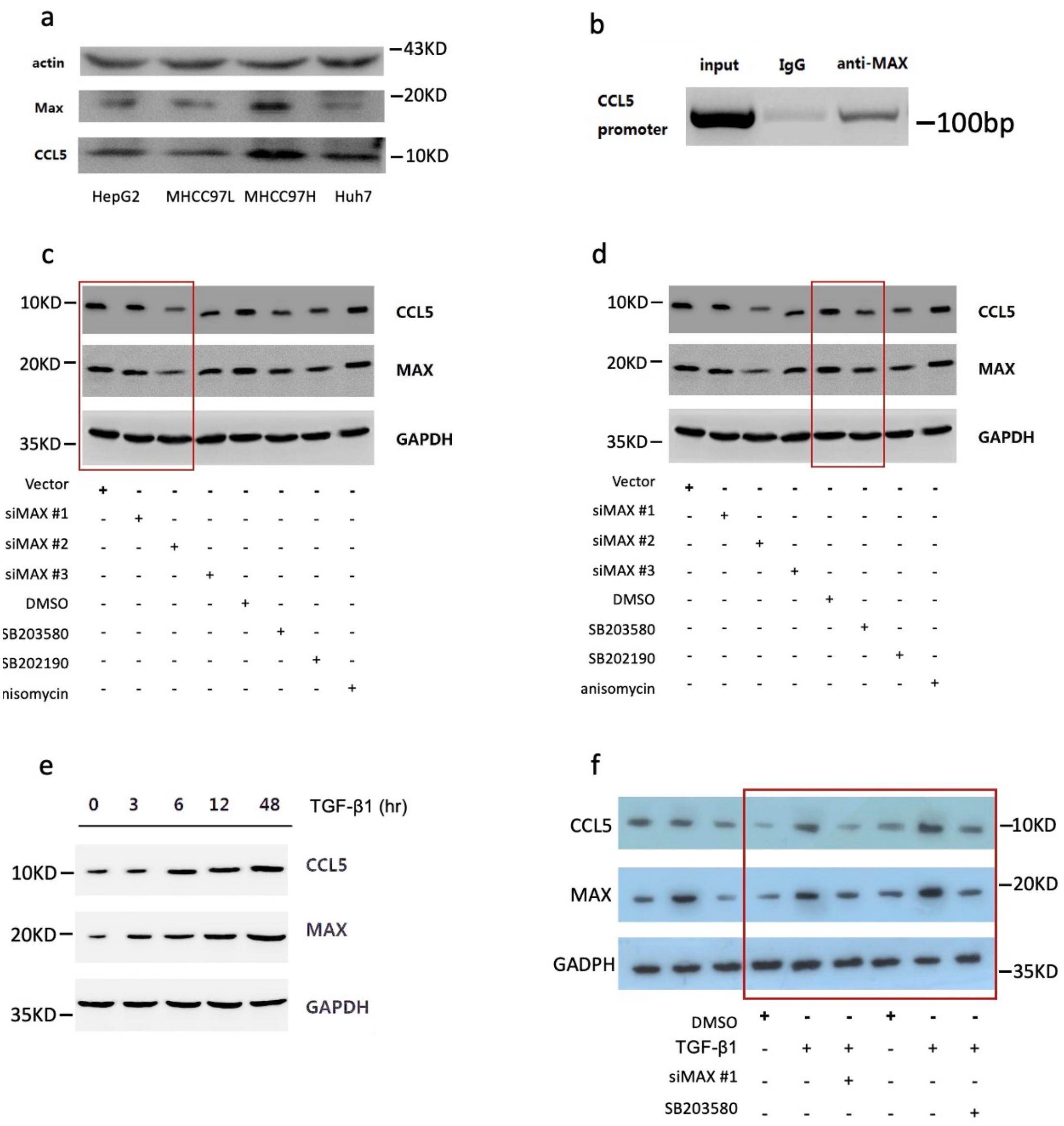

Supplementary Figure 7. Full western blot images. **a** Full western blot image of Figure 5a. **b** Full western blot image of Figure 6b. **c** Full western blot image of Figure 6c. **d** Full western blot image of Figure 6d. **e** Full western blot image of Supplementary Figure 6l. **f** Full western blot image of Figure 6h.
