# Supplementary material for: Dissecting spatial heterogeneity and the immune-evasion mechanism of CTCs by single-cell RNA-seq in hepatocellular carcinoma
Source: Nat Commun. 2021 Jul 2;12:4091. doi: 10.1038/s41467-021-24386-0 (PMC8253833; doi:10.1038/s41467-021-24386-0)
Supplement: Supplementary file 2 — Description of Additional Supplementary Files [file 41467_2021_24386_MOESM2_ESM.docx]

**Description of Additional Supplementary Files**

File Name: Supplementary Data 1

Description: Clinical information of HCC patients enrolled for CTC scRNA-seq.

File Name: Supplementary Data 2

Description: Information of samples and sequencing data.

File Name: Supplementary Data 3

Description: Genes list and pathway enrichments result of CTCs up-regulated genes from primary tumor.

File Name: Supplementary Data 4

Description: Genes list and pathway enrichments result for differential genes between neighboring vascular compartments.

File Name: Supplementary Data 5

Description: Clinical information of HCC patients in two independent validation cohorts.

File Name: Supplementary Data 6

Description: Univariate and multivariate cox proportional regression analysis of factors associated with time to recurrence and overall survival.
