# Supplementary material for: Dissecting spatial heterogeneity and the immune-evasion mechanism of CTCs by single-cell RNA-seq in hepatocellular carcinoma
Source: Nat Commun. 2021 Jul 2;12:4091. doi: 10.1038/s41467-021-24386-0 (PMC8253833; doi:10.1038/s41467-021-24386-0)
Supplement: Supplementary file 3 — Supplementary Data 1 [file 41467_2021_24386_MOESM3_ESM.pdf]

Supplementary Table 1.

Clinical information of HCC patients enrolled for CTC scRNA-seq.

Patient	Etiology	Cirrhosis	Tumor no.	Diameter, cm	Vascular invasion	BCLC stage	AFP, ng/ml
P1	HBV	No	1	18	Yes	B	28.5
P2	HBV	Yes	2	6	Yes	B	37.8
P3	HBV	No	1	11	Yes	B	179.4
P4	HCV	Yes	1	5.5	Yes	A	4.9
P5	HBV	Yes	1	10	Yes	B	13,775
P6	HBV	Yes	1	7	Yes	B	9.6
P7	HBV	Yes	1	5.5	Yes	A	7.1
P8	HBV	Yes	1	17	Yes	B	141.7
P9	HBV	Yes	1	6	No	A	68.2
P10	HBV	No	1	6.5	No	A	10.1

Note: The listed diameter is the maximum diameter of the largest lesion. Abbreviations: AFP, alpha-fetoprotein; BCLC, Barcelona Clinic Liver Cancer; HBV, hepatitis B virus; HCV, hepatitis C virus.
