# Supplementary material for: Dissecting spatial heterogeneity and the immune-evasion mechanism of CTCs by single-cell RNA-seq in hepatocellular carcinoma
Source: Nat Commun. 2021 Jul 2;12:4091. doi: 10.1038/s41467-021-24386-0 (PMC8253833; doi:10.1038/s41467-021-24386-0)
Supplement: Supplementary file 7 — Supplementary Data 5 [file 41467_2021_24386_MOESM7_ESM.pdf]

Supplementary Data 5.

Clinical information of HCC patients in two independent validation cohorts.

Characteristic	Validation cohort 1 (n = 27)	Validation cohort 2 (n = 83)
Age, years		
≤50	7	39
>50	20	44
Gender		
Male	22	64
Female	5	19
HBsAg		
Negative	4	10
Positive	23	73
HCV		
Negative	27	81
Positive	0	2
Liver cirrhosis		
No	6	19
Yes	21	64
AFP, ng/mL		
≤20	12	43
>20	15	40
Tumor number		
Single	17	74
Multiple	10	9
Tumor size, cm		
≤5	15	60
>5	12	23
Tumor encapsulation		
No	16	34
Yes	11	49
Vascular invasion		
No	18	51
Yes	9	32
Edmondson stage		
I-II	16	59
III-IV	11	24
BCLC stage		
0-A	16	70
B+C	11	13
Staging of liver cancer in China (2017 edition)		
Ia+Ib	14	72
IIa+IIb	10	6
III	3	5

Note: the listed diameter is the maximum diameter of the largest lesion. Abbreviations: AFP, alpha-fetoprotein; BCLC, Barcelona Clinic Liver Cancer; HBsAg, surface antigen of hepatitis B; HCV, hepatitis C virus IF, immunofluorescence.
