# Supplementary material for: Dissecting spatial heterogeneity and the immune-evasion mechanism of CTCs by single-cell RNA-seq in hepatocellular carcinoma
Source: Nat Commun. 2021 Jul 2;12:4091. doi: 10.1038/s41467-021-24386-0 (PMC8253833; doi:10.1038/s41467-021-24386-0)
Supplement: Supplementary file 8 — Supplementary Data 6 [file 41467_2021_24386_MOESM8_ESM.pdf]

Supplementary Data 6. Univariate and multivariate cox proportional regression analysis of factors associated with time to recurrence and overall survival

	Time to Recurrence				Overall Survival			
	Univariate Analysis		Multivariate Analysis		Univariate Analysis		Multivariate Analysis	
Variables	HR (95% CI)	*P*	HR (95% CI)	*P*	HR (95% CI)	*P*	HR (95% CI)	*P*
Age (>50y *vs*. ≤50y)	0.57 (0.27–1.19)	0.136	N.A.	N.A.	0.70 (0.29–1.68)	0.419	N.A.	N.A.
Gender (male *vs*. female)	0.77 (0.38–4.13)	0.717	N.A.	N.A.	0.86 (0.31–2.35)	0.761	N.A.	N.A.
HBsAg (Positive *vs*. Negative)	0.97 (0.34–2.77)	0.947	N.A.	N.A.	0.59 (0.17–2.04)	0.409	N.A.	N.A.
Liver cirrhosis (Yes *vs*. No)	0.95 (0.40–2.23)	0.907	N.A.	N.A.	1.77 (0.52-6.04)	0.326	N.A.	N.A.
AFP (>20 ng/ml *vs*. ≤20 ng/ml)	1.37 (0.66-2.85)	0.402	N.A.	N.A.	2.27 (0.91-5.71)	0.079	N.A.	N.A.
Tumor number (Multiple *vs*. Single)	2.80 (1.14-6.90)	0.025	3.61 (1.44-9.04)	0.006	0.92 (0.21-3.96)	0.909	N.A.	N.A.
Tumor size (>5 cm *vs*. ≤5 cm)	1.74 (0.82-3.68)	0.149	N.A.	N.A.	1.21 (0.47-3.15)	0.339	N.A.	N.A.
Microvascular invasion (Yes *vs*. No)	3.09 (1.46-6.57)	0.003	2.25 (1.02-4.92)	0.044	2.80 (1.14-6.87)	0.024	2.25 (0.88-5.78)	0.091
Edmondson stage (III-IV *vs*. I-II)	1.66 (0.78-3.51)	0.627	N.A.	N.A.	1.05 (0.40-2.73)	0.920	N.A.	N.A.
BCLC stage (B+C *vs*. 0+A)	3.60(0.85-9.34)	0.001	N.A.	N.A.	1.47 (0.49-4.40)	0.491	N.A.	N.A.
CNLC stage (II+III *vs*.I)	3.74 (1.65-8.51)	0.002	N.A.	N.A.	1.87 (0.62-5.61)	0.449	N.A.	N.A.
Balance between CCL5+ CTC and circulating Tregs (IV vs I+II+III)	4.25 (2.00-9.04)	<0.001	3.71 (1.67-8.22)	0.001	2.62 (1.08-6.32)	0.032	2.03 (0.80-5.14)	0.134
Balance between CCL5+ CTC and circulating Tregs (III+IV vs I+II)	4.61 (1.19-5.74)	0.017	0.88 (1.67-8.22)	0.871	1.57 (0.64-3.85)	0.320	N.A.	N.A.

Clinicopathological variables were adopted for their prognostic significance by univariate analyses. Because BCLC and CNLC stages were associated with the clinical categories of tumor characteristics, liver function and performance status, it was not included in multiple analyses to avoid potential bias.

Abbreviations: CTC, circulating tumor cell; PV, peripheral vein; PA, peripheral artery; HV, hepatic vein; PoV, portal vein; HBsAg, hepatitis B surface antigen; AFP, alpha-fetoprotein; BCLC, Barcelona Clinic Liver Cancer, CNLC, China Liver Cancer Staging, N.A., not applicable

Group I: $Treg^{low}$/$CCL5^{+}$ CTC^{low}; Group II: $Treg^{low}$/$CCL5^{+}$ CTC^{high}; Group III: $Treg^{high}$/$CCL5^{+}$ CTC^{low}; Group IV: $Treg^{high}$/$CCL5^{+}$ CTC^{high}
