# Supplementary material for: Dissecting spatial heterogeneity and the immune-evasion mechanism of CTCs by single-cell RNA-seq in hepatocellular carcinoma
Source: Nat Commun. 2021 Jul 2;12:4091. doi: 10.1038/s41467-021-24386-0 (PMC8253833; doi:10.1038/s41467-021-24386-0)
Supplement: Supplementary file 9 — Reporting Summary [file 41467_2021_24386_MOESM9_ESM.pdf]

nature research

Corresponding author(s): Xin-Rong Yang

Last updated by author(s): Jan 31, 2021

Reporting Summary

Nature Research wishes to improve the reproducibility of the work that we publish. This form provides structure for consistency and transparency in reporting. For further information on Nature Research policies, see our Editorial Policies and the Editorial Policy Checklist.

Statistics

For all statistical analyses, confirm that the following items are present in the figure legend, table legend, main text, or Methods section.

n/a	Confirmed	
☐	☒	The exact sample size (*n*) for each experimental group/condition, given as a discrete number and unit of measurement
☐	☒	A statement on whether measurements were taken from distinct samples or whether the same sample was measured repeatedly
☐	☒	The statistical test(s) used AND whether they are one- or two-sided<br>*Only common tests should be described solely by name; describe more complex techniques in the Methods section.*
☒	☐	A description of all covariates tested
☐	☒	A description of any assumptions or corrections, such as tests of normality and adjustment for multiple comparisons
☐	☒	A full description of the statistical parameters including central tendency (e.g. means) or other basic estimates (e.g. regression coefficient) AND variation (e.g. standard deviation) or associated estimates of uncertainty (e.g. confidence intervals)
☐	☒	For null hypothesis testing, the test statistic (e.g. *F*, *t*, *r*) with confidence intervals, effect sizes, degrees of freedom and *P* value noted<br>*Give P values as exact values whenever suitable.*
☒	☐	For Bayesian analysis, information on the choice of priors and Markov chain Monte Carlo settings
☒	☐	For hierarchical and complex designs, identification of the appropriate level for tests and full reporting of outcomes
☐	☒	Estimates of effect sizes (e.g. Cohen's *d*, Pearson's *r*), indicating how they were calculated

Our web collection on statistics for biologists contains articles on many of the points above.

Software and code

Policy information about availability of computer code

Data collection	No software for data collection was used
Data analysis	BWA (v0.7.17), SAMtools (v1.7), Picard (v2.18.0), Ginkgo (http://qb.cshl.edu/ginkgo/), SOAPnuke (v1.5.0), TopHat (v2.0.12), edgeR (v3.28.1), RSEM (v1.2.31), Rtsne (v0.15), Monocle (v1.6.2), GSEA (v3.0), inferCNV(v1.2.1), etc. were used in the analysis. The key scripts were provided online at https://github.com/Cacti-Jiang/CTC. Flow cytometry data was analyzed using BD FACSDiva (v8.0.1) and FlowJo (v10). The BD cytometric bead array (CBA) data was analyzed using BD FCAP array software (v1.0). Multiplex immunofluorescence staining assay were analyzed by HALO Software (v 3.0, Indica Labs)

For manuscripts utilizing custom algorithms or software that are central to the research but not yet described in published literature, software must be made available to editors and reviewers. We strongly encourage code deposition in a community repository (e.g. GitHub). See the Nature Research guidelines for submitting code & software for further information.

Data

Policy information about availability of data

All manuscripts must include a data availability statement. This statement should provide the following information, where applicable:

- Accession codes, unique identifiers, or web links for publicly available datasets
- A list of figures that have associated raw data
- A description of any restrictions on data availability

All data generated for this study is available through European Genome-phenome Archive (accession ID: EGAS00001005204) and the CNGB Nucleotide Sequence Archive (GNSA, https://db.cngb.org/cnsa/, CNP0000095)

Field-specific reporting

Please select the one below that is the best fit for your research. If you are not sure, read the appropriate sections before making your selection.

- [x] Life sciences
- [ ] Behavioural & social sciences
- [ ] Ecological, evolutionary & environmental sciences

For a reference copy of the document with all sections, see nature.com/documents/nr-reporting-summary-flat.pdf

Life sciences study design

All studies must disclose on these points even when the disclosure is negative.

Sample size	No statistical methods were used to predetermine sample sizes. For in vivo studies, Sample sizes were selected empirically from our previous experimental experience with similar assays (Yang WJ, 2020, PMID: 33093445; Chen XX, 2018, PMID: 30305612). In our experience, n=5 mice per group is sufficient to detect meaningful biological difference with good reproducibility
Data exclusions	No exclusion of data was made.
Replication	Experiments were performed at least 3 times and/or with sufficient cells/animals per group to demonstrate statistical significance. All attempts at replication were successful.
Randomization	For cell experiments, all cells in each experiment were from the same pool of parental cells. For in vivo experiments, mice were randomly allocated into cages before treated with corresponding cells or antibodies.
Blinding	For data collected by objective instruments, such as CTC analyzer, qPCR cyclers, microscopy software, FACS, animal CT systems, and western blotting, the investigators were not blinded to group allocation during data collection. However, the investigator was blinded at the time of data analysis. Laboratory personnel were blind to animal randomization to drug treatment, which was performed by the PI. However, laboratory personnel could not be blinded during the experiment as they needed to know which groups to treat with which drugs. However, the laboratory personnel was blinded during the data analysis from each individual mice. Sequencing data analyses was performed by the Biostatistian who were blinded to patient information and experimental groups. Pathological examination was done in a blinded way.

Reporting for specific materials, systems and methods

We require information from authors about some types of materials, experimental systems and methods used in many studies. Here, indicate whether each material, system or method listed is relevant to your study. If you are not sure if a list item applies to your research, read the appropriate section before selecting a response.

Materials & experimental systems

n/a	Involved in the study
[ ]	[x] Antibodies
[ ]	[x] Eukaryotic cell lines
[x]	[ ] Palaeontology and archaeology
[ ]	[x] Animals and other organisms
[ ]	[x] Human research participants
[x]	[ ] Clinical data
[x]	[ ] Dual use research of concern

Methods

n/a	Involved in the study
[x]	[ ] ChIP-seq
[ ]	[x] Flow cytometry
[x]	[ ] MRI-based neuroimaging

Antibodies

Antibodies used

CTC immunofluorescence staining assay
PE-labeled EpCAM (130-098-113; Miltenyi Biotec), Alexa-594 labeled pan-CK (628606; BioLegend), Alexa-594 labeled CK19 (ab203443; Abcam), FITC labeled CD45 (304006; BioLegend), APC labeled CD45 (304012; BioLegend), Alexa-488 labeled CCL5 (IC278G-100UG; R&D Systems).

Chromatin Immunoprecipitation:
anti-MAX (ab53570, Abcam), anti-IgG (Cat # ab6715, Abcam)

Western blot:
anti-GAPDH (sc-47724, Santa Cruz), anti-CCL5 (2988S, Cell Signaling Technology), anti-MAX (4739S, Cell Signaling Technology),

Immunohistochemistry assay:
anti-FoxP3 (Cat# ab20034, diluted 1:250, abcam), anti-CCL5 (Cat# AF-278-NA, diluted 1:250, R&D Systems), anti-FOXP3 (Cat# 2A11G9, diluted 1:200, Santa Cruz), anti-CD8 (Cat# 6A242, diluted 1:200, Santa Cruz) and anti-Granzyme B (Cat# sc8022, diluted 1:250, Santa Cruz).

Multiplex immunofluorescence staining assay:
anti-CK8/18 (Cat# BX50145, Biolynx), anti-FoxP3 (Cat# MAB8214, R&D systems), anti-CCL5 (Cat# AF-278-NA, R&D systems), anti-CD8 (Cat# BX50036-C3, Biolynx), anti-CCR5 (Cat# MAB182-100, R&D systems), anti-CD3 (Cat# BX50022, Biolynx), anti-CD45RO (Cat# GM074202, Gene Tech), horseradish peroxidase-conjugated secondary antibody (1:1, Cat# DS9800, Lecia Biosystems); horseradish peroxidase-conjugated secondary antibody (Cat# A10011-6, WiSee Biotechnology), horseradish peroxidase-conjugated secondary antibody (Cat# A10012-6, WiSee Biotechnology)

In vitro assay:
anti-CCL5 (Cat# MAB678, R&D systems) and control IgG (Cat# 1-001-A, R&D systems)

In vivo assay:
InVivoMAb anti-mouse CD25 (Cat# BE0012, Rat IgG1, BioXcell)
InVivoMAb rat IgG1 isotype control (Cat# BE0088, Rat IgG1 BioXcell)

Validation

The following antibodies were quality-checked and validated based on the information provided on the manufacturers' websites:
-PE-labeled EpCAM antibody: https://www.biolegend.com/en-us/products/alexa-fluor-594-anti-cytokeratin-pan-reactive-antibody-12687
-Alexa-594 labeled CK19 antibody: https://www.abcam.cn/alexa-fluor-594-cytokeratin-19-antibody-ep1580y-ab203443.html
-FITC labeled CD45: https://www.biolegend.com/en-us/products/fitc-anti-human-cd45-antibody-707
-APC labeled CD45: https://www.biolegend.com/en-us/products/apc-anti-human-cd45-antibody-705
-Alexa-488 labeled CCL5: https://www.rndsystems.com/cn/products/human-ccl5-rantes-alexa-fluor-488-conjugated-antibody-21445_ic278g
-annti-MAX: https://www.abcam.cn/max-antibody-73c5a-ab53570.html
-anti-IgG: https://www.abcam.cn/rabbit-human-igg-hl-ab6715.html
-anti-GAPDH: https://www.scbt.com/p/gapdh-antibody-0411?requestFrom=search
-anti-CCL5: https://www.cellsignal.cn/products/primary-antibodies/rantes-p20-antibody/2988?site-search-type=Products&N=4294956287&Ntt=2988s&fromPage=plp&_requestid=302671
-anti-MAX: https://www.cellsignal.cn/products/primary-antibodies/max-s20-antibody/4739?site-search-type=Products&N=4294956287&Ntt=4739s&fromPage=plp&_requestid=302712
-anti-FoxP3: https://www.abcam.cn/foxp3-antibody-236ae7-ab20034.html
-anti-CCL5: https://www.rndsystems.com/cn/products/human-ccl5-rantes-antibody_af-278-na
-anti-FOXP3: https://www.scbt.com/p/foxp3-antibody-2a11g9?requestFrom=search
-anti-CD8: https://www.scbt.com/p/cd8-alpha-antibody-6a242?requestFrom=search
-anti-Granzyme B: https://www.scbt.com/zh/p/granzyme-b-antibody-2c5?requestFrom=search
-anti-CK8/18 http://www.biolynx.cn/products/methods/112/138/501.html
-anti-CD8 http://www.biolynx.cn/products/methods/112/138/306.html
-anti-CD3 http://www.biolynx.cn/products/methods/112/138/292.html
-anti-FoxP3: https://www.rndsystems.com/cn/products/human-mouse-foxp3-antibody-1054c_mab8214
-anti-CCR5: https://www.rndsystems.com/cn/products/human-ccr5-antibody-45531_mab182
-anti-CCL5: https://www.rndsystems.com/cn/products/human-primate-ccl5-rantes-antibody-21418_mab678
-anti-CD45RO https://www.genetech.com.cn/goods/goods_detail/262.html
-horseradish peroxidase-conjugated secondary antibody: https://shop.leicabiosystems.com/us/ihc-ish/detection-systems/pid-bond-polymer-refine-detection
-horseradish peroxidase-conjugated secondary antibody: http://www.yuanxibio.cn/productinfo/90811.html
-horseradish peroxidase-conjugated secondary antibody: http://www.yuanxibio.cn/productinfo/90801.html
-IgG isotype control: https://www.rndsystems.com/cn/products/normal-human-igg-control_1-001-a
-InVivoMAb anti-mouse CD25 antibody: https://bxcell.com/product/m-cd25/
-InVivoMAb rat IgG1 isotype control (Clone HRPN, Rat IgG1 BioXcell)

Eukaryotic cell lines

Policy information about cell lines

Cell line source(s)

Hepa1-6, Hun7 and Hepa3B cell lines were obtained from Shanghai Cell Bank, Chinese Academy of Sciences. MHCC97H (highly metastatic human HCC cell lines) and MHCC97L (Low metastatic human HCC cell lins) were established at our institute

Authentication

The cell line was characterized by the cell bank based on cell morphology, post-freeze viability, isoenzyme analysis, DNA fingerprinting analysis, mycoplasma contamination testing, and bacterial and fungal contamination.

Mycoplasma contamination

All cell lines tested negative for mycoplasma contamination

Commonly misidentified lines
(See ICLAC register)

None of cell lines used in this study are listed by ICLAC

Animals and other organisms

Policy information about studies involving animals; ARRIVE guidelines recommended for reporting animal research

Laboratory animals	Animals used in in vivo experiments were four- to six-week-old male C57BL/6J mice from Lingchang Biotech Shanghai. Animals were housed at approximately 22±2ºC, humidity 50±10% on a 12-hour light, 12-hour dark cycle.
Wild animals	This study did not involve any wild animals
Field-collected samples	No field-collected samples were used in this study
Ethics oversight	All experiments were reviewed and approved by Zhongshan Hospital Animal Care and Use Committee

Note that full information on the approval of the study protocol must also be provided in the manuscript.

Human research participants

Policy information about studies involving human research participants

Population characteristics	The demographics of the patients enrolled in this study are provided in Table S1 and S2
Recruitment	From September 2013 to June 2016, a total of 120 patients newly diagnosed with HCC undergoing curative resection were recruited to this study. The patient recruitment criteria were: (1) definitive pathological or radiological diagnosis according to American Association for Study of Liver Disease guidelines, (2) no extrahepatic metastasis at the time of diagnosis, and (3) no prior anticancer treatment. All blood samples were anonymized before being processed in laboratory. Therefore, self-selection bias resulting from recruitment was less likely.
Ethics oversight	Ethical approval for the use of human subjects was obtained from the Research Ethics Committee of Zhongshan Hospital. Informed written consent was obtained from each patient.

Note that full information on the approval of the study protocol must also be provided in the manuscript.

Flow Cytometry

Plots

Confirm that:

- [x] The axis labels state the marker and fluorochrome used (e.g. CD4-FITC).
- [x] The axis scales are clearly visible. Include numbers along axes only for bottom left plot of group (a 'group' is an analysis of identical markers).
- [x] All plots are contour plots with outliers or pseudocolor plots.
- [x] A numerical value for number of cells or percentage (with statistics) is provided.

Methodology

Sample preparation	Human and mouse peripheral blood samples were collected and red blood cells were removed using ACK lysis buffer (Gibco)
Instrument	Aria II flow cytometer
Software	BD DIVA software used for collection, FlowJo software used for analysis
Cell population abundance	No cell sorting was performed in this manuscript
Gating strategy	Singlets were gated according to the pattern of FSC-H vs. FSC-A. Positive populations were determined by the specific antibodies, which were distinct from negative populations.

- [x] Tick this box to confirm that a figure exemplifying the gating strategy is provided in the Supplementary Information.
